# Covalent ferrocene conjugation as an intramolecular strategy for photostability in fluorescein

**DOI:** 10.1038/s41598-025-34817-3

**Published:** 2026-01-07

**Authors:** Gilbert K. Kosgei, P. U. Ashvin Iresh Fernando, Harley R. Mcalexander, Afrachanna D. Butler

**Affiliations:** 1 US Army Engineer Research and Development Center, Environmental Laboratory, 3909 Halls Ferry Road, Vicksburg, MS 39180 USA; 2 US Army Engineer Research and Development Center, Cold Regions Research and Engineering Laboratory, 72 Lyme Road, Hanover, NH 03755 USA; 3 SIMETRI, Inc., 937 S Semoran Blvd Suite 100, Winter Park, FL 32792 USA

**Keywords:** Photostability, Ferrocene conjugates, Fluorescein, Photoinduced electron transfer (PET), Redox chemistry, Intramolecular quenching, Chemistry, Materials science

## Abstract

Photobleaching severely limits the utility and long-term reliability of fluorescence-based measurements. To address this long-standing limitation, we synthesized a conjugate that covalently links fluorescein isothiocyanate (FITC) to ferrocene (Fc), a redox-active metallocene. Our two-step synthesis involved reduction of ferrocene methylene azide followed by formation of a stable thiourea linkage. Photophysical characterization confirmed highly efficient intramolecular quenching, evidenced by an 81.5% reduction in quantum yield (Φ) and a shortened lifetime (τ = 3.2 ns vs. 4.1 ns for FITC). The Fc–FITC conjugate exhibited an 11-fold increase in photobleaching half-life (693 vs. 63 min for FITC), retaining 94% of its initial fluorescence after 60 min of constant 23 mW/cm^2^ irradiation, compared to only 52% for FITC. Direct singlet oxygen (^1^O_2_​) quantification using Singlet Oxygen Sensor Green (SOSG) confirmed that Fc conjugation reduces the photosensitization rate to only 28% of that of native FITC. Sodium azide (NaN_3_​) quenching assays further validated the suppression of reactive oxygen species (ROS), as the Fc–FITC system exhibited negligible quenching (4.6%) compared to the significant response of native FITC (32.5%). This stabilization arises from a Photoinduced Electron Transfer (PET) mechanism that suppresses formation of the destructive triplet state (T^1^​). A quantitative Rehm–Weller analysis (ΔG_PET_ ≈ − 0.76 eV) and direct ROS validation establish a robust mechanistic basis for this photoprotective effect. Together, these findings establish a unique intramolecular photostabilization strategy where signal durability and quantitative precision are prioritized over peak brightness, offering a framework for designing robust hybrid redox–fluorophore probes suited for persistent sensing and long-term quantitative analysis.

**Supplementary Information:**

The online version contains supplementary material available at 10.1038/s41598-025-34817-3.

## Introduction

Fluorescence-based techniques are essential across modern science, driving advancements in biological imaging, sensing, diagnostics, and functional materials design^1–6^. These applications rely on synthetic organic chromophores that emit light upon excitation^7,8^. However, the utility of these fluorophores is severely limited by photobleaching, an irreversible photochemical degradation that undermines fluorescence intensity and measurement reliability during prolonged experimentation^9–12^. This degradation is also a critical concern in optoelectronic technologies, such as organic solar cells, where light-induced degradation limits operational lifetime^13,14^.

Photobleaching occurs when sustained light exposure leads to the irreversible degradation of the fluorophore, distinguishing it from reversible processes like blinking^11,15^. The underlying process involves complex interactions between excited fluorophores and molecular oxygen^12^. This typically generates long-lived triplet excited states (T^1^​) and reactive oxygen species (ROS), leading to structural alterations and the irreversible loss of optical function^14–18^. To counter this photobleaching, two conventional strategies have historically been employed. The first involves the use of external scavengers, such as Trolox (water-soluble derivative of vitamin E) and ascorbic acid, which are chemical additives that act after triplet states or ROS have already formed. While widely used, these methods typically yield modest enhancements of two- to five-fold in half-life, suffer from rapid consumption, and often interferes with biological systems^19,20^. The second approach employs passive intramolecular strategies, which attempt to stabilize the chromophore by physical means, such as molecular rigidification or encapsulation^9^. Despite these efforts, the challenge remains unresolved for high-sensitivity and long-term applications. Our strategy for mitigation involves the active intramolecular strategy, which involves the covalent integration of a photoprotective moiety into the chromophore architecture. This approach is necessary to provide inherent, non-sacrificial, long-term stability and is known to achieve significantly higher enhancement factors than external additives^21,22^.

Ferrocene (Fc) is a compelling scaffold due to its robust organometallic structure, low toxicity, high stability, and well-defined redox behavior^23–25^. Ferrocene’s ability to undergo efficient reversible one-electron oxidation to the ferrocenium cation enables it to quench excited states and dissipate excess energy via Photoinduced Electron Transfer (PET) and triplet-state quenching mechanisms^26,27^. Our results confirm that this active strategy yields a photostability enhancement factor of 11-fold, a performance result significantly exceeding that of typical external scavengers. This capability directly enhances chromophore stability by mitigating photodegradation, a principle supported by its application in porphyrin-based photosensitizers^28,29^. While ferrocene–fluorophore conjugates are widely developed for molecular sensing applications^25^, including the multichannel systems^30,^ ferrocene–fluorescein chemosensors^31^, and for chemically-triggered probes such as the hypochlorous acid (HOCl)-responsive constructs^32^, these systems typically leverage the Fc unit as a redox-active reporter or switch. Thus, these modulated signals depend on specific binding or redox events. In contrast, the present work employs the ferrocene moiety for a fundamentally different purpose: active intramolecular photoprotection. Rather than modulating fluorescence output, in response to an external analyte, the Fc unit in our design serves as a constant, built-in energy sink that dissipates excited-state energy through PET and suppresses triplet-state formation, thereby mitigating photobleaching under continuous illumination.

Fluorescein and its derivative fluorescein isothiocyanate (FITC) exemplify the problem; while widely used in diverse applications such as real-time quantitative assays, single-molecule microscopy, and cellular sensing due to its high quantum yield and favorable biocompatibility^33–35^, it is highly susceptible to photobleaching, which compromises its performance in quantitative assays^11,36–41^. Covalent conjugation of a redox-active protective unit to FITC is a promising, yet underexplored, approach that leverages the principle of “self-healing”, where a covalently linked redox modulator mitigates photobleaching internally^22^^,^^21^. Analogous systems have shown notable improvements by quenching triplet states^27,42^. This design concept is particularly relevant for applications like single-molecule imaging, where prolonged observation is crucial^43^.

We hypothesized that the covalent linkage of ferrocene to FITC would significantly enhance the photostability of the fluorophore, thereby extending its functional lifetime in demanding analytical and bio-based applications. To test this, we executed a modular synthetic route to generate a novel Fc-FITC conjugate. The conjugates were extensively characterized, and comparative photobleaching studies were conducted under continuous illumination in an aqueous-based system. This study presents a reliable framework for synthesizing redox-active, photostabilized fluorophore systems, establishing a pathway for mitigating fluorophore photodegradation through rational molecular design validated by direct singlet oxygen (^1^O_2_) quantification, suited for persistent sensing and long-term quantitative analysis.

## Results and discussion

### Synthesis of ferrocene-fluorescein conjugate (Fc-FITC)

**Fig. 1:**
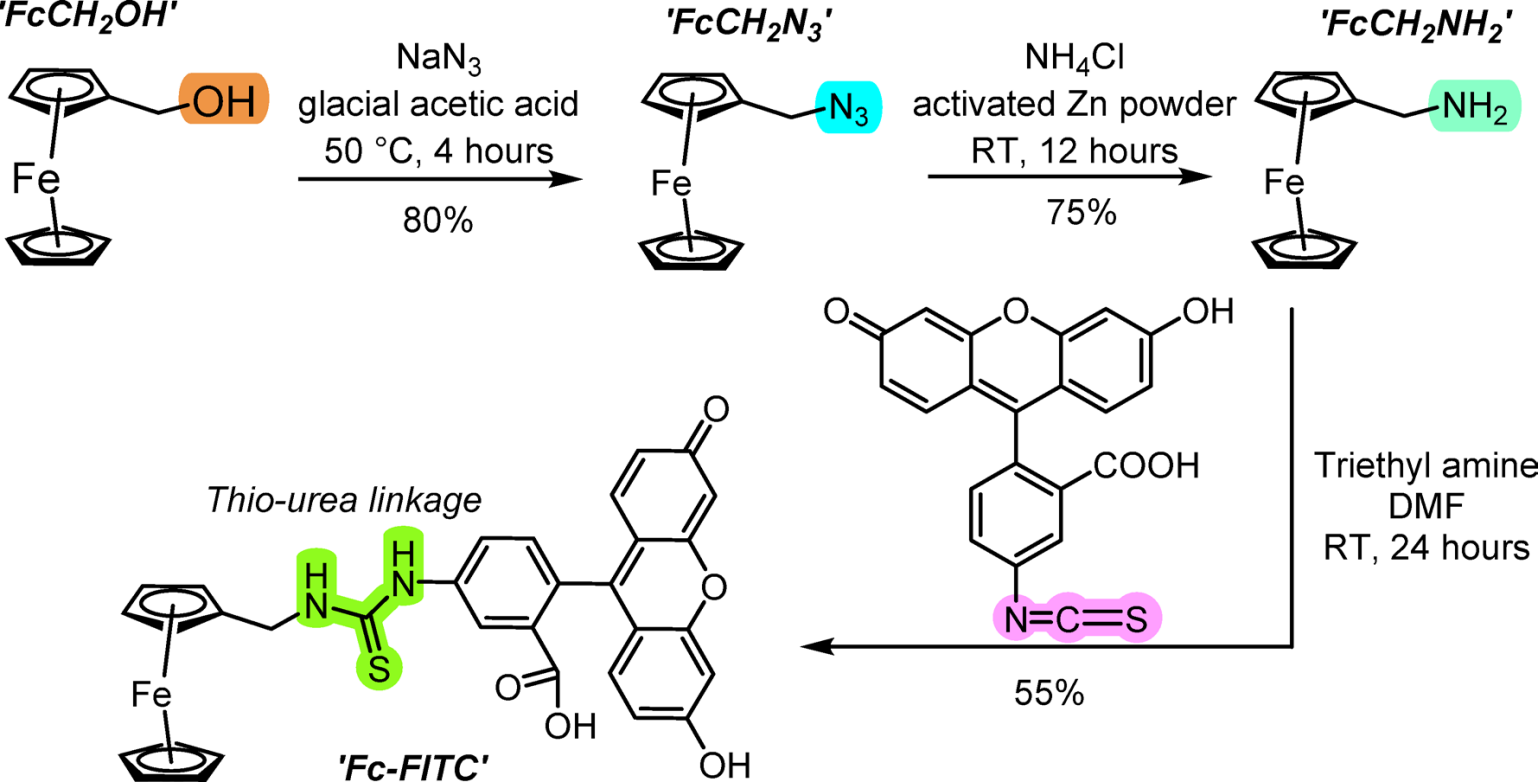
Complete synthesis scheme to obtain Fc-FITC conjugate. The diagram illustrates the three-step synthesis. The highlighted area represents the key functional group being transformed, and the percent yield is shown for each reaction step.

The Fc-FITC conjugate was successfully synthesized via a three-step pathway (Fig. 1). The initial steps focused on functionalizing the ferrocene scaffold to yield the key intermediate FcCH_2_NH_2_​. Comprehensive spectroscopic data (^1^H NMR, ^13^C NMR, FTIR, and HRMS) confirmed the stepwise transformation of all intermediates, as detailed in the Supporting Information (SI). The final step involved the conjugation of FcCH_2_NH_2_​ ​ with FITC using triethylamine in anhydrous DMF to form the final Fc-FITC conjugate via a stable thiourea linkage. This specific linkage was chosen because the inherent robustness of the C − N bond ensures that any observed photostability is attributable to the electronic interaction between Fc and FITC, rather than the stability of the linker itself.

Successful formation of the conjugate was verified by spectroscopic analysis. Mass spectrometry provided strong evidence, detecting the deprotonated molecular ion at m/z = 603.0716, which closely matched the calculated value (Figure S25). Furthermore, ^1^H NMR spectroscopy exhibited characteristic signals for both the Fc and FITC moieties, including two highly deshielded proton signals (∼10.2 ppm and ∼8.3 ppm) that confirmed the thiourea bond formation (Figure S23). It is relevant to mention that these two amide groups are chemically different because each is attached to two different molecules on the either side of the thio-urea linker. This successful synthesis and characterization set the stage for evaluating the conjugate’s photophysical properties and photostability.

### Photophysical properties of ferrocene analogs

**Fig. 2:**
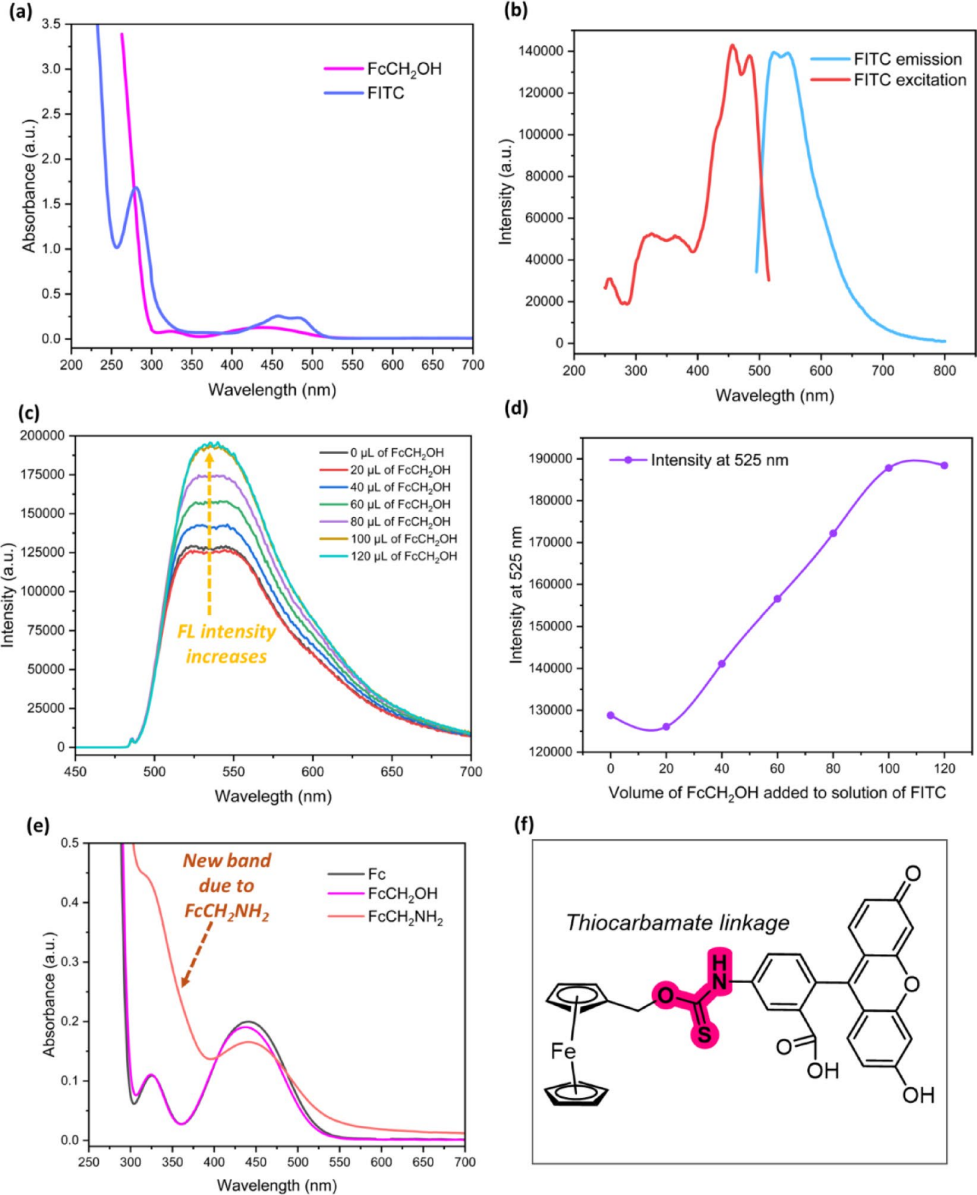
Photophysical characterization and intermolecular interaction of FITC and ferrocene analogs. (**a**) UV–Vis absorption of FcCH_2_OH and FITC in methanol. (**b**) FITC excitation (red, λ_em_​=525 nm, red) and emission (blue, λ_ex​=_485 nm) spectra. (**c**,**d**) Quenching anomaly: Rising fluorescence emission (**c**) and its linear 2D plot (**d**) of FITC upon addition of a dilute solution of FcCH_2_OH (1.34 mmol) in methanol. (**e**) UV–Vis absorption spectra comparing Fc, FcCH_2_OH, and FcCH_2_NH_2_​​ in ethanol. (**f**) Proposed chemical structure of the O-thiocarbamate intermediate. All spectra recorded at concentration of ~ 2 µM (A < 0.2) to ensure accuracy.

The absorption spectra of the ferrocene derivatives (Fc, FcCH_2_OH, and FcCH_2_NH_2_​​), recorded in ethanol, are presented in Fig. 2e. FcCH_2_OH (selected as the starting material and close analog for the Fc unit) provides the baseline for two characteristic absorption bands: a lower-energy band observed at ∼440 nm attributed to the metal-to-ligand charge transfer (MLCT) or d − d transition^44,45^, and a higher-energy *π*→*π*∗ transition near ∼330 nm (a minor peak, see Figs. 2a and S26).

The spectral effects caused by the substituents in the intermediate FcCH_2_NH_2_​​ demonstrate a key electronic difference. The electron-donating amine group enhances the absorption intensity near ∼330 nm, confirming the successful conversion from the hydroxyl group. Furthermore, the amine substituent causes a slight redshift of the 440 nm band^44,45^, consistent with the electron-donating nature of the − NH_2_​ group, making the Fc core slightly easier to oxidize. These results demonstrate that substituent electronic effects strongly influence the photophysical properties of ferrocene derivatives, enabling potential tuning of their electronic behavior.

### Photophysical properties of FITC chromophore

Establishing the fundamental photophysical properties, including absorption, emission, and quantum yield, is critical for analyzing how fluorophores like FITC undergo photobleaching^9,46^. The FITC chromophore displays a prominent *π*→*π*∗ transition, characterized by an absorption band at 495 nm (Figs. 2b and S26). Its high molar extinction coefficient (∼75,000 M^−1^cm^−1^) signifies the strong light absorption necessary for efficient fluorescence. FITC is well-known for its highly efficient fluorescence emission at ∼525 nm, with a reported high quantum yield (Φ_F_​) of about 0.92 in 0.1 NaOH^47^. As expected, the optical properties of an equimolar FcCH_2_OH and FITC mixture would be dominated by the FITC chromophore. The key properties of individual FITC compared to Fc are summarized in Table S2.

Intriguingly, initial investigations in methanol revealed an unexpected phenomenon: an apparent increase in FITC emission upon the addition of dilute FcCH_2_OH (Fig. 2c and d). This finding was unusual, as ferrocene derivatives are established fluorescence quenchers, and this anomaly may stem from the surrounding solvent’s polarity and viscosity influencing photophysical behavior^48,49^. A further possibility for this anomaly is that the hydroxyl group of FcCH_2_OH reacts with FITC via nucleophilic addition, forming an O-thiocarbamate or thionocarbamate ester (Fig. 2f). The electronic properties of this ester linkage would differ from the thiourea, potentially causing the observed emission increase, a change previously shown to provide additional stabilization against photo-oxidation^50^. To mitigate these potential solvent or pH-induced artifacts, we conducted subsequent investigations in buffered aqueous solution. Initial buffer stability studies at varying concentrations are shown in Figure S28.

### pH optimization

The defining characteristic of fluorescein derivatives, including FITC, is their existence in multiple prototropic forms (e.g., neutral, monoanionic, and dianionic), whose distribution is highly dependent on environmental pH^51–53^. This dynamic equilibrium greatly influences absorption and emission, with the highly fluorescent dianionic form predominating at basic pH conditions^54^.

To optimize conditions for accurate Fc-FITC characterization, we examined FITC’s pH-dependent spectral properties in an EtOH/H_2_​O (1:1 v/v) mixture across a broad pH range (2 to 13, Table S1). The composite results illustrating these pH-dependent transitions are presented in Fig. 4. Ethanol was chosen over methanol to minimize evaporation and ensure volume consistency during measurements.

Plots of UV-Vis absorption at 500 nm and emission intensity at 530 nm against pH revealed an inflection point (detailed spectra in Fig. 4a and c). Fitting these curves (Fig. 4e and f) allowed us to determine the pKa of FITC to be 5.39 (averaged from absorption and emission titration curves). This value is notably lower than the commonly reported p*K*a​ of ∼6.4 for FITC in pure water^55^, a downward shift likely attributed to the reduced dielectric constant of the mixed solvent, which alters the ionization behavior^51,53^. Since maximum FITC emission was observed in the pH 8–9 range within this solvent system, a pH of 8 was ultimately selected for subsequent quantitative fluorescence studies. This choice provides a crucial balance, ensuring enhanced signal intensity while remaining within the physiologically relevant window required for many bio-based applications. Initial buffer stability results at varying concentrations are shown in Figure S28.

### Intermolecular quenching studies

Following the anomalous observations in methanol, the interaction between Fc and FITC was re-evaluated in the controlled buffered system (EtOH/ H_2_O, 1:1 v/v, pH 8). In this stabilized environment, Fc consistently behaved as an excited-state fluorescence quencher, confirming its electron-donating nature and ability to participate in the requisite excited-state quenching mechanisms. This result highlights the critical importance of using buffered solutions for accurate assessment of intermolecular interactions.

To quantify this interaction further, Stern–Volmer experiments were conducted by incrementally adding Fc derivatives to FITC solutions prepared in the pH 8 buffer. The data analyzed using the linear Stern–Volmer equation (F₀/F = 1 + KSV[Q]) yielded plots (Figure S27) that exhibited strong linearity (*R*^2^ > 0.96), characteristic of a dynamic quenching mechanism.

The quenching constants (*K*_*SV*_​) were determined and ranked as follows (Table S3): Fc (1030 M^−1^) > FcCH_2_NH_2_ ​(170 M^−1^) > FcCH_2_OH (119 M^−1^).The FITC (non-functionalized) fluorescence lifetime (*τ₀*​) was determined experimentally to be 4.1 ns, under identical matched solvent and pH conditions (Section S6), and the corresponding bimolecular quenching rate constants (*k*_*q*_ *​= K*_*SV*_*​/ τ₀*​) were calculated, with Fc exhibiting the highest efficiency (*k*_*q*​_ = 2.51 × 10^11^ M^−1^s^−1^), suggesting its most effective electron transfer to the FITC excited state^56^. The significantly lower *k*_*q*_​ values for the functionalized ferrocenes (e.g., FcCH_2_NH_2_ at 17% relative to Fc) suggest that steric hindrance and the electronic effects of the substituents modulate the quenching efficiency. The unsubstituted Fc moiety’s significantly higher *K*_*SV*_ meant that the required linear quenching response was achieved at a reduced overall concentration compared to the other derivatives. These results are consistent with PET as the predominant mechanism for quenching and suggest that conjugation may be a viable strategy for stabilizing the fluorophore.

### Photophysical properties of Fc-FITC conjugate

With the photophysical characteristics of the individual Fc and FITC components established, we focused on the Fc-FITC conjugate to assess the impact of the covalent linkage and resulting intramolecular interactions.

**Fig. 3:**
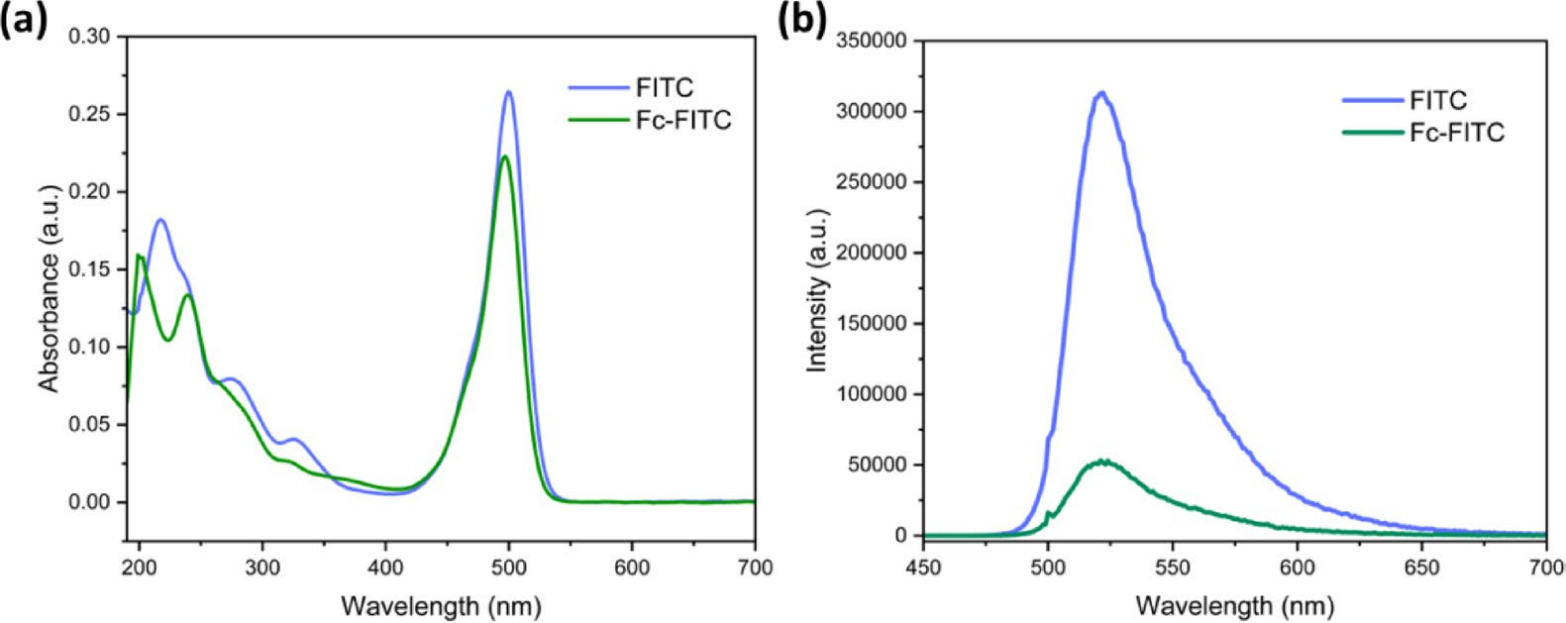
Comparative photophysical spectra of FITC and Fc-FITC (1:1 v/v, EtOH/H_2_O, pH 8 buffer). (**a**) UV-Vis absorption spectra. (**b**) Relative emission spectra (λ_ex_​=490 nm), showing fluorescence quenching upon conjugation. All spectra recorded at concentration of ~ 2 µM (A < 0.2) to ensure accuracy.

The Fc-FITC conjugate exhibited a strong absorption band at 497 nm, nearly identical in position to free FITC (Figs. 3a and S29). However, the conjugate showed notable changes in the higher-energy region (280–330 nm), suggesting a modification in FITC’s electronic structure upon conjugation via the thiourea linkage, potentially due to electron delocalization or charge transfer interactions with the ferrocene unit. Upon excitation at 490 nm, the Fc-FITC conjugate displayed fluorescence emission at 525 nm (Fig. 3b), similar to free FITC. However, the intensity of this emission was significantly reduced, providing initial evidence that the Fc moiety acts as an efficient intramolecular quencher even when covalently linked.

The most striking evidence of intramolecular interaction was the determined fluorescence quantum yield (Φ_F_​) of the Fc-FITC conjugate, which was 0.17. This represents a substantial 81.5% reduction compared to the Φ_F_​ of free FITC (0.92)^57–59^, indicating highly efficient intramolecular excited-state quenching by the ferrocene moiety. Further supporting this effect, the fluorescence lifetime of Fc-FITC was measured via time-correlated single-photon counting (TCSPC) to be 3.2 ns which is shorter than the measured 4.1 ns for free FITC under identical matched conditions (Figs. 7d and S30). This lifetime shortening provides direct kinetic evidence of the quenching process; the PET mechanism introduces a rapid, highly efficient decay pathway (*k*_PET_​) that increases the molecule’s total decay rate (*k*_*total*_). Since lifetime (*τ*) is inversely proportional to *k*_*total*_​, i.e., (*τ* = 1/*k*_total_​), the introduction of this fast PET pathway results in an immediate and significant reduction in the time the molecule spends in the vulnerable singlet state (S^1^​). The reduced quantum yield and shortened lifetime are strong indicators that the excited-state energy is rapidly dissipated through a non-radiative competitive decay pathway, consistent with PET, a mechanism observed in other ferrocene-chromophore systems^26,27^.

### pH-dependent behavior and pKa determination

**Fig. 4:**
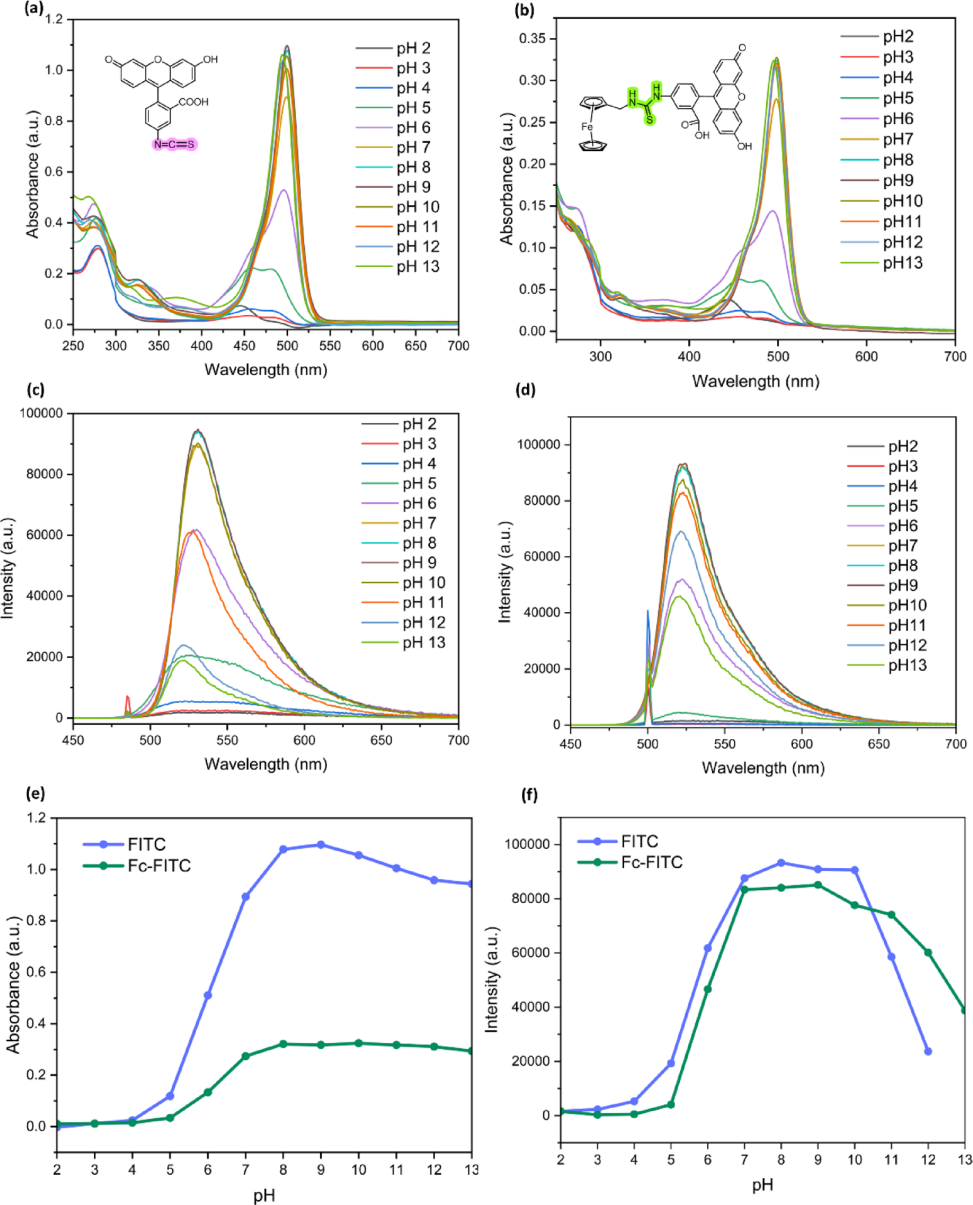
pH dependence of FITC and Fc-FITC in 1:1 v/v EtOH/H_2_O buffer. Panels (**a**) and (**b**) show UV-Vis absorption spectra for FITC and Fc-FITC, respectively (pH 2 − 13). Panels (**c**) and (**d**) show corresponding fluorescence emission spectra. The bottom panels, (**e**) absorption at 500 nm and (**f**) emission at 530 nm vs. pH, were used to plot the pKa​ titration curves for both compounds. All initial spectra recorded at concentration of ~ 2 µM (A < 0.2) to ensure accuracy.

Understanding the pH dependence of Fc-FITC is crucial, as pH significantly influences FITC’s spectral properties and stability. Fluorescence emission measurements were conducted across the pH range of 2–13 (Fig. 4b and d).

From the Fc-FITC titration curves (Fig. 4e and f), the p*K*a​ of the conjugate was determined to be 5.78 (averaged from absorption and emission data). This represents a ∼0.4 unit upward shift in p*K*a​ compared to free FITC (pKa​ = 5.39) in the same mixed solvent system (EtOH/H_2_​O). This shift suggests that ferrocene conjugation moderately stabilizes the protonated state or alters the local microenvironment, thereby influencing FITC’s acid-base equilibrium. The highest peak emission intensity for Fc-FITC was observed within the pH 7–10 range, mirroring the behavior of free FITC. Although Fc-FITC demonstrated higher emission intensity compared to FITC at more alkaline pH levels (pH 11–13), suggesting potentially improved stability in that range, a pH of 8 was ultimately selected for subsequent photobleaching studies. The selected pH 8 provides a crucial compromise, ensuring enhanced signal intensity while remaining within the physiologically relevant window required for biological applications.

### Electrochemistry

**Fig. 5:**
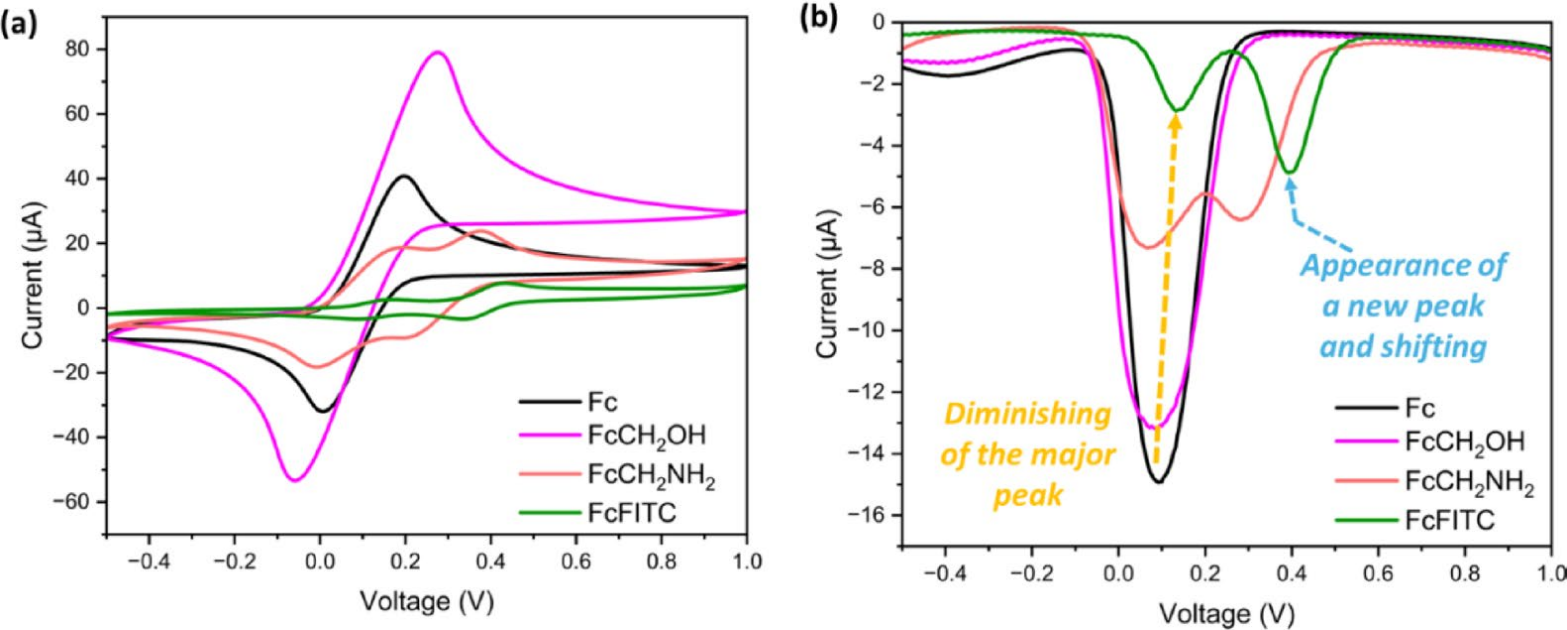
Comparative electrochemical analysis of ferrocene derivatives. (**a**) CV and (**b**) DPV of Fc, FcCH_2_OH, FcCH_2_NH_2_​, and the Fc-FITC conjugate. (anhydrous CH₃CN/0.1 M TBAPF₆, scan at a 100 mV/s from + 1.0 V to − 0.5 V). The working electrode was glassy carbon electrode (GCE), the counter electrode was platinum (Pt) wire, and the reference electrode was Ag/Ag+. The half-wave potential (E_1/2_​) represents the potential halfway between the anodic peak (E_p.a._) for oxidation and the cathodic peak (E_pc_) for reduction. The separation peak (ΔE_p_​) represents the difference between E_p.a._ and E_pc_.

Understanding the redox behavior of ferrocene is critical, as its reversible oxidation influences energy dissipation and suppresses the long-lived triplet state that causes photobleaching. We therefore performed cyclic voltammetry (CV) and differential pulse voltammetry (DPV) based experiments on Fc, its synthetic intermediates, and the final Fc-FITC conjugate. All measurements utilized a conventional three-electrode system in anhydrous acetonitrile (CH_3_​CN) (containing 0.1 M TBAPF_6​_ to assess intrinsic redox processes accurately)^23^. The measured half-wave potentials (*E*_1/2_​) and characteristics are summarized in Table 1 and illustrated in the comparative voltammograms (Fig. 5).

**Table 1:** Electrochemical data for ferrocene derivatives and the Fc-FITC conjugate. *DPV measurements were used to estimate the value of E_1/2_.

Compound	Redox process (Fc/Fc^+^)	*E_1/2_​ (V vs. Ag/Ag^+^)	E_*p*.a._​ (V vs. Ag/Ag^+^)	E_pc​_ (V vs. Ag/Ag^+^)	ΔE_*p*_​ (mV)	​I_pc_/I_*p*.a._
Ferrocene (Fc)	Fc/Fc^+^	0.09	0.2	0.01	190	0.785
FcCH_2_OH	Fc/Fc^+^	0.09	0.27	-0.06	210	0.675
FcCH_2_NH_2_	Fc/Fc^+^	0.06	0.19	0.01	180	0.972
	Secondary	0.28	0.39	0.21	180	0.381
Fc-FITC	Oxidation (minor)	0.13	0.17	0.09	80	1.315
	Oxidation (major)	0.39	0.44	0.34	100	0.446

The CV data (Fig. 5a) for the intermediates showed expected electronic effects. Unsubstituted Fc and FcCH_2_OH both exhibited an *E*_1/2_​ of + 0.09 V, indicating the hydroxymethyl substituent has minimal electronic influence^23^. In contrast, the electron-donating amine group in FcCH_2_NH_2_ ​ made the ferrocene core easier to oxidize, shifting *E*_1/2_​ to a lower potential of + 0.06 V. The secondary oxidation wave observed for FcCH_2_NH_2_​ at + 0.28 V is assigned to the oxidation of the pendant - NH_2_​ substituent itself^60^.

We acknowledge that the observed large Δ*E*_*p*​_ values (80–210 mV) indicate quasi-reversible, non-ideal behavior, which is common in substituted ferrocene derivatives due to adsorption and functional group kinetics^60^, as well as intramolecular hydrogen bonding interactions which are typical of Fc-thiourea systems. However, the primary objective of our CV analysis was not to assess kinetic reversibility (Δ*E*_*p*_​) or the number of electrons (n), but rather to accurately determine the thermodynamic oxidation potential (E_ox_​). This potential is the crucial input required for calculating the Rehm–Weller ΔG_PET​_ driving force, and the data is valid for this specific mechanistic analysis. The Fc-FITC conjugate, however, displayed two distinct, reversible oxidation peaks in both CV and the more sensitive DPV measurements (Fig. 5b). Crucially, the major oxidation peak for Fc-FITC is at a significantly higher potential of + 0.39 V. This significant anodic shift (relative to unconjugated Fc at + 0.09 V) provides clear evidence of strong electronic coupling between the electron-donor Fc and the electron-acceptor FITC moiety^23,61–63^.

The dominant oxidation peak at + 0.39 V vs. Ag/Ag + is accordingly assigned to the quasi-reversible Fc/Fc^+^ couple, strongly shifted anodically by the electron-withdrawing thiourea–fluorescein framework. This assignment is consistent with ferrocenyl acyl-thiourea derivatives^64^, where electron-withdrawing substituents shift the Fc/Fc^+^ couple. In contrast, the irreversible electrochemical oxidation of thiourea^65^ typically occurs at significantly higher potentials and exhibits an irreversible voltametric signature that lacks the clear return (reduction) peak observed in our system. This intramolecular electronic communication is a well-known feature of ferrocene-containing systems where the linker influences electron transfer, as seen in studies on molecular wires, mixed-valent compounds, and other ferrocene-chromophore conjugates^25,44^. Slight deviations from ideal reversibility may be observed, likely due to adsorption or coupled chemical interactions with N-H groups of the thio-urea linkaged^60^. While a minor peak at + 0.13 V may be attributed to an oxidation event related to the thiourea sulfur group^23,61,63,66,67^, the significant shift strongly reinforces that the conjugation successfully tunes the electrochemical properties of ferrocene, enabling its vital role as the PET donor in the photoprotective mechanism.

### Computational studies

To gain deeper insights into the photophysical behavior and photostabilization mechanism, we performed computational studies on both the deprotonated FITC and Fc-FITC conjugate (to mimic experimental conditions). Our goal was to simulate their electronic excitation (UV-Vis) spectra using Time-Dependent Density Functional Theory (TDDFT). Full details on the functionals (B3LYP and CAM-B3LYP), basis sets, and parameters are given in Section S7 in the SI. The optimized ground-state conformations for both molecules are shown in Fig.ure S31.

The calculations showed notable variations in the HOMO–LUMO energy gaps (Δ*E*_*gap*_) depending on the functional used and whether a solvent model was included (Table 2). DFT analysis of the Fc-FITC conjugate revealed a crucial separation of frontier orbital density. For the photoprotective PET mechanism to be energetically viable, electron transfer must proceed from HOMO(Fc) to the excited-state LUMO(FITC*), forming the Fc+/FITC*^−^ charge separated state.

**Table 2:** HOMO-LUMO gaps analysis for FITC and Fc-FITC highlights solvation effects.

Condition	FITC HOMO-LUMO (E_gap_​) kcal/mol	Fc-FITC HOMO-LUMO (E_gap_​) kcal/mol	Gap change HOMO-LUMO (≈ΔE_gap_​) kcal/mol
B3LYP//gas	56.9	36.6	−20.3
B3LYP//SMD	69.1	69.5	+ 0.4
CAM-B3LYP//gas	110.4	85.5	−24.9
CAM-B3LYP//SMD	120.1	120.5	+ 0.4

The SMD-solvated results (Table 2) showed that the Δ*E*_gap_​ for FITC and Fc-FITC remained very similar upon conjugation (Δ*E*_gap​_ ≈ +0.4 kcal/mol) (Figures S32–S35), consistent with our experimental observation that the main absorption peak for Fc-FITC (497 nm) is nearly identical to unconjugated FITC (495 nm)^44^. This preservation of the FITC energy gap confirms it is the active light absorber, restoring the correct donor–acceptor alignment for PET.

**Fig. 6:**
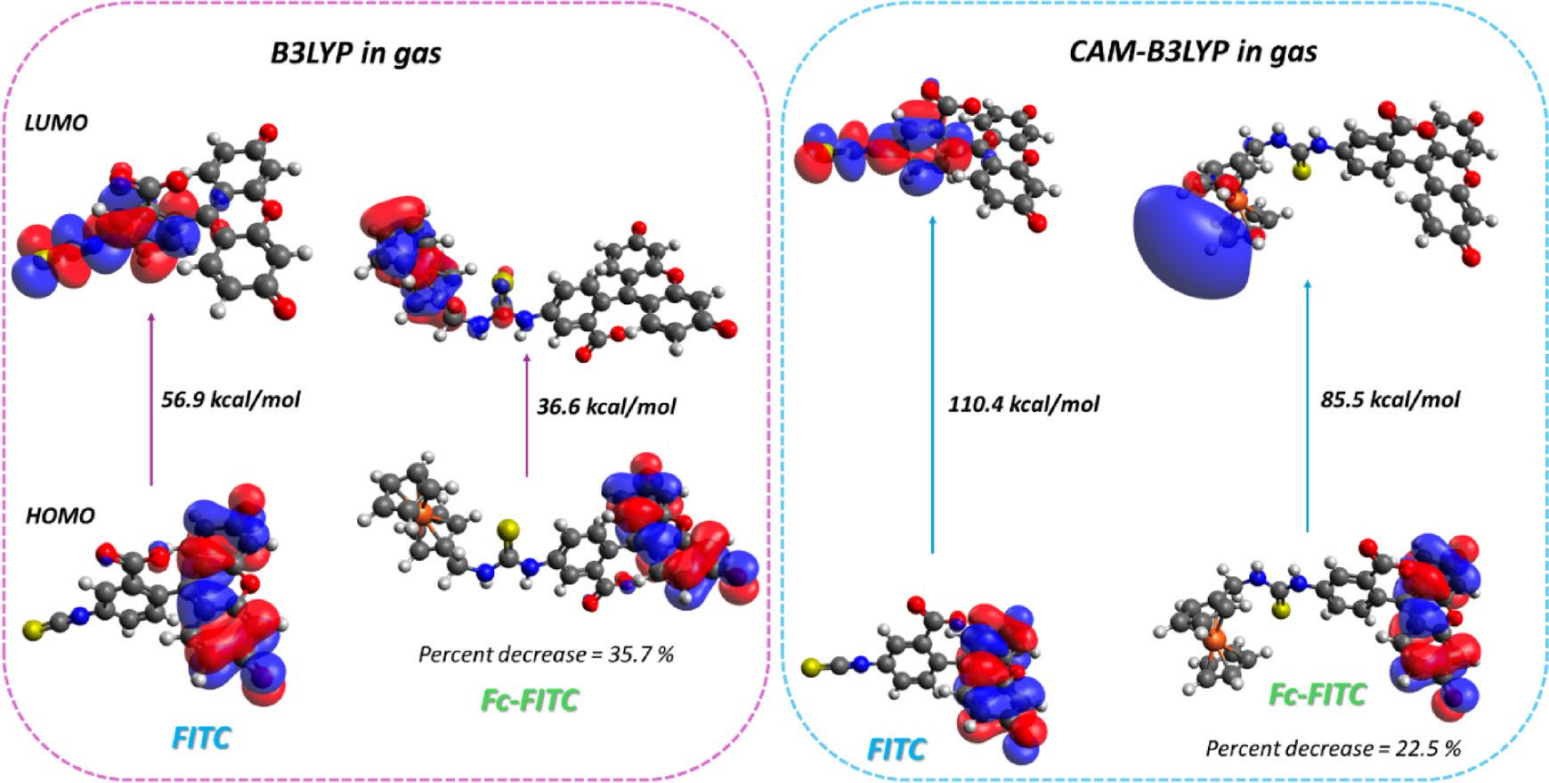
DFT predictions of the frontier molecular orbitals for FITC (left) and Fc-FITC (right). The bottom energy levels represent the HOMO, and the top levels represent the LUMO.

In contrast, the gas-phase calculations showed a dramatic decrease in the gap upon conjugation (up to Δ*E*_gap​_ ≈ −25 kcal/mol) (Fig. 6 and Figures S36–S37). This highlights the crucial role of the solvent environment: the strong interactions between the polar solvent and the charged fluorophore screen the electronic coupling, ensuring the system operates through the efficient intra-moiety Fc→FITC* PET channel under experimental conditions. Furthermore, Fc conjugation introduces new, accessible LUMO levels associated with the Fc moiety (Fig. 6). These low-lying orbitals likely contribute to photostabilization by acting as an efficient destination for back electron transfer (BET) from the FITC*^−^ radical anion, accelerating the non-radiative (NR) return to the ground state (GS).

### Photostability studies

We assessed the long-term performance of the Fc-FITC conjugate under continuous high-intensity irradiation (*λ*_ex_​ = 320–500 nm, 23 mW/cm^2^, pH 8), building upon the kinetic evidence that ferrocene conjugation resulted in an 81.5% reduction in fluorescence quantum yield (Φ_F_​) and a shortened fluorescence lifetime (3.2 ns). These preliminary findings strongly suggested efficient excited-state quenching of the FITC chromophore by the redox-active ferrocene unit.

**Fig. 7:**
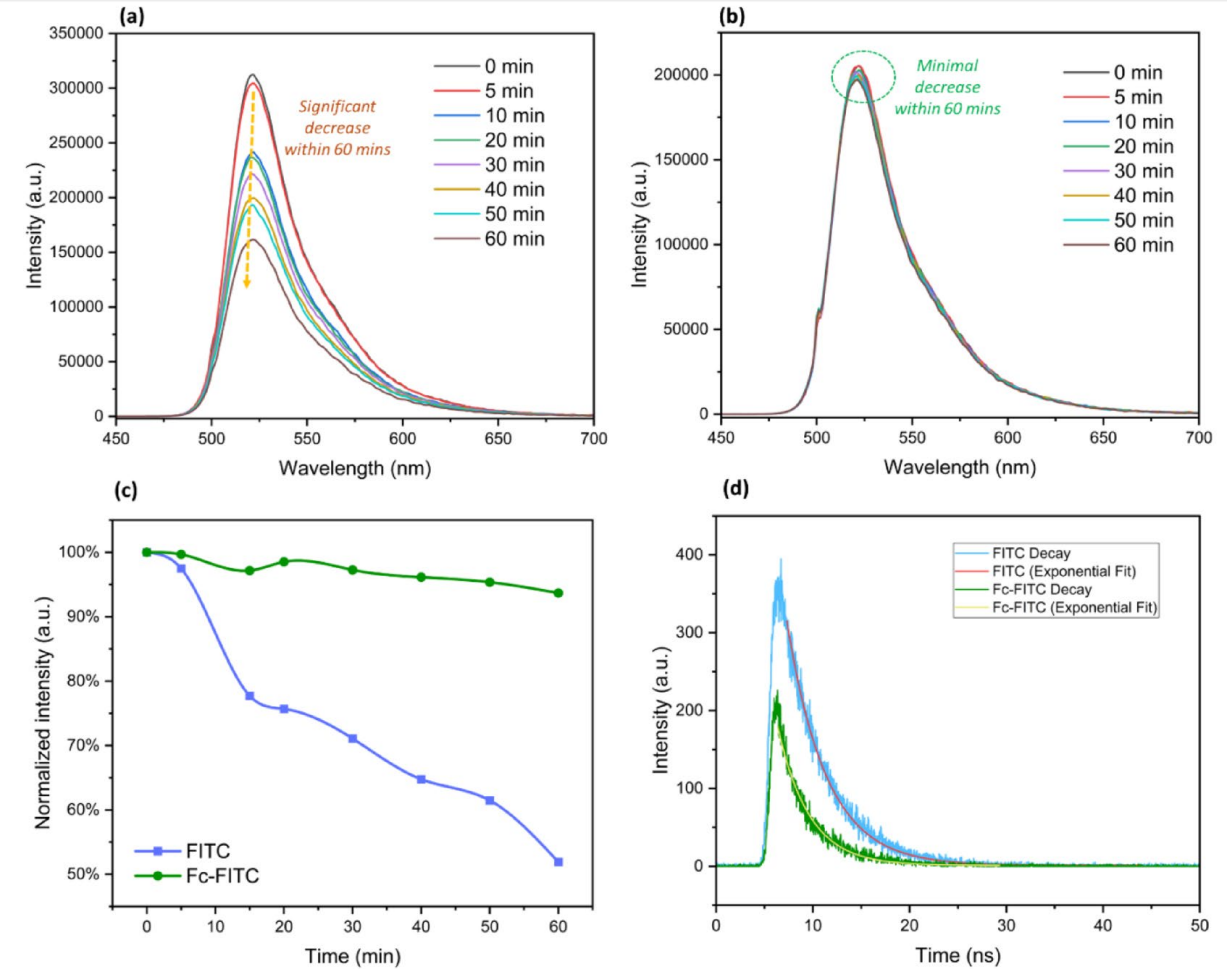
Comparative photostability and fluorescence dynamics of FITC and Fc-FITC. The time-resolved emission spectra collected over 60 min of continuous UV-Vis irradiation (320–500 nm, 23 mW/cm^2^ in 1:1 EtOH/H_2​_O pH 8 buffer) show the photobleaching kinetics for (**a**) FITC and (**b**) Fc-FITC. (**c**) Comparative photobleaching curves showing normalized emission intensity at 530 nm versus irradiation time; and (d) Fluorescence decay plot for FITC & Fc-FITC (λ_ex​=_247 nm).

As quantified in Fig. 7c and shown in the time-resolved spectra of Fig. 7a and b, unconjugated FITC exhibited pronounced photobleaching, with its normalized emission intensity falling to approximately 52% over the 60-minute exposure period. In stark contrast, the Fc-FITC conjugate demonstrated enhanced photostability, retaining approximately 94% of its initial fluorescence, despite its lower intrinsic quantum yield. While FITC initially provides a higher photon count, its 48% signal loss over a single hour creates significant quantitative uncertainty and requires frequent recalibration; in contrast, the 94% retention of Fc-FITC offers the stable, horizontal baseline required for precise long-term sensing.

We note that the decay observed under continuous illumination deviates from linearity on a semi-logarithmic scale, exhibiting clear multi-phase kinetics. This non-ideal behavior is characteristic of xanthene dyes, where photobleaching is governed by sequential T^1^​ state accumulation and subsequent reaction with oxygen, generating ROS that initiates multistep degradation pathways^9,11,16^. The exponential fit was therefore applied primarily to extract a comparative t_½_ value as a robust metric for evaluating the 11-fold enhancement provided by the Fc conjugation.

Fitting the fluorescence decay data to a first-order exponential model (*I(t) =* I_0_· *e*^*− Kt*^), the photobleaching half-life (and *t*_1/2_ = ln (2)/*k*) was determined. The *t*_*1/2*_ of FITC was only 63 min (*k*_*FITC*_ *≈ 0.011 min*^*−1*^), while the Fc-FITC conjugate displayed a remarkably prolonged *t*_*1/2*_ of 693 min (*k*_*Fc−FITC*_ *≈ 0.001 min*^*− 1*^). This study confirms that ferrocene conjugation leads to an 11-fold increase in the photobleaching half-life, a result that significantly outperforms the two- to five-fold enhancements typically achieved using external scavengers^19,20^ effectively mitigating the photodegradation of the FITC chromophore (Figure S38 and Table S4). The visual comparison of the FITC and Fc-FITC solutions before and after irradiation further confirms the mitigation of degradation (Figure S39).

###  Thermodynamic feasibility

To assess the thermodynamic driving force for the proposed PET from Fc to the excited FITC* acceptor, we performed a quantitative Rehm–Weller analysis, which estimates the change in Gibbs free energy (Δ*G*_PET_​) using the equation^68^:

$$\Delta {G_{{\mathrm{PET}}}}\left( {{\mathrm{eV}}} \right) = {E_{ox}}\left( {{\mathrm{D}}/{{\mathrm{D}}^ + }} \right) - {E_{red}}\left( {{\mathrm{A}}/{{\mathrm{A}}^ - }} \right) -{E_{0 - 0}} + C$$

Due to the nonaqueous reference (Ag/Ag^+^ in CH_3_​CN), we recalibrated the measured oxidation potential to the standard calomel electrode (SCE) using the Fc/Fc + couple as an internal standard^69^. The Fc–FITC oxidation potential (+ 0.39 V vs. Ag/Ag^+^) was converted via:

$$\begin{aligned} {E_{{\mathrm{ox}}}} \left( {{\mathrm{D}}/{{\mathrm{D}}^ + }} \right) & = + 0.{\text{39 V }} - 0.{\mathrm{29}}~{\text{V }}\left( {{\mathrm{Ag}}/{\mathrm{A}}{{\mathrm{g}}^ + }~{\mathrm{vs}}~{\mathrm{Fc}}/{\mathrm{F}}{{\mathrm{c}}^ + }} \right) \\ & \quad + 0.{\mathrm{4}}0~{\text{V }}\left( {{\mathrm{Fc}}/{\mathrm{Fc}} + ~{\mathrm{vs}}~{\mathrm{SCE}}} \right) = + 0.{\mathrm{5}}0~{\mathrm{V}}~{\mathrm{vs}}~{\mathrm{SCE}} \end{aligned}$$

The acceptor reduction potential of deprotonated FITC was taken from literature as *E*_*red*_​(A/A^−^) ≈ − 1.21 V vs. SCE^70^. The singlet excited-state energy (E_0 − 0_ ​= 1240/503 nm ≈ 2.47 eV) was determined from the intersection of normalized excitation and emission spectra (*λ*_0−0_​ = 503 nm). The Coulombic term (*C ≈ 0*) is negligible in our polar solvent system. Substituting into the Rehm–Weller equation:

$$\Delta {G_{{\mathrm{PET}}}} = \left( { + 0.{\mathrm{5}}0~{\mathrm{V}}} \right) - \left( { - {\mathrm{1}}.{\mathrm{21}}~{\mathrm{V}}} \right) - \left( {{\mathrm{2}}.{\mathrm{47}}~{\mathrm{eV}}} \right) = - 0.{\mathrm{76}}~{\mathrm{eV}}$$

This corresponds to − 73.3 kJ kJ mol^−1^ (1 eV ≈ 96.485 kJ mol^−1^, is the conversion factor) indicating that PET from Fc to FITC* is thermodynamically favorable (exergonic). This result provides the crucial quantitative foundation for the observed 11-fold photostability enhancement and supports the observed suppression of ROS by confirming that the protective PET pathway is thermodynamically favored over the destructive intersystem crossing (ISC) pathway. This result provides strong quantitative support for the proposed PET mechanism as the core deactivation pathway, explaining the observed photostability enhancement.

### Photostabilization mechanism

The 11-fold enhancement in photostability confirms the hypothesis that the ferrocene moiety acts as an efficient photoprotective agent, primarily through intramolecular PET and energy dissipation.

The mechanism is driven by the significant 81.5% quenching of FITC’s fluorescence and the shortened lifetime (*τ* = 3.2 ns), providing direct kinetic evidence of a fast, non-radiative decay pathway introduced by the covalent linkage. When FITC is excited to the singlet state (S^1^​), the Fc unit introduces a competitive PET decay pathway via the thiourea linkage, providing an efficient route for rapid deactivation^16^. This fast process effectively suppresses the intersystem crossing (ISC) (S^1^​→T^1^​) pathway, minimizing the formation and lifetime of the long-lived triplet state (T^1​^ ​). Since T^1​^ ​ is highly susceptible to reactions that generate ROS like singlet oxygen (^1^O_2_), which are the primary cause of irreversible chemical degradation (photobleaching)^16,38,71^, its suppression directly correlates with the enhanced photostability^50,72^. In this work, we experimentally validated the PET decay pathway via comparative ROS generation studies (singlet oxygen validation section). The observed 11-fold half-life enhancement is directly supported by a ~ 72% reduction in the rate of singlet oxygen sensitization, providing a clear macroscopic link between PET-mediated triplet quenching and chemical durability.

This PET mechanism is strongly supported by both electrochemical and computational results: Electrochemical feasibility is shown by CV and DPV data, which confirm that Fc undergoes reversible oxidation. The dramatic anodic shift of its redox potential upon conjugation indicates that Fc readily acts as an electron donor to the excited FITC*, enabling efficient Fc→FITC* PET^3^. The computational results are validated by the B3LYP//SMD calculations, which confirm that in the solvated environment, the initial light absorption is localized on the FITC core (FITC→FITC*). The suppression of strong electronic coupling by the SMD model demonstrates how the polar solvent screens the charge, thereby forcing the system to operate through the highly efficient Fc→FITC* PET channel. Furthermore, Fc conjugation introduces new, accessible LUMO levels associated with the Fc moiety (Fig. 6) that were unavailable in native FITC. These low-lying orbitals likely contribute to photostabilization by acting as an efficient destination for BET from the FITC∗− radical anion, accelerating the non-radiative (NR) return to the ground state (GS).

**Fig. 8:**
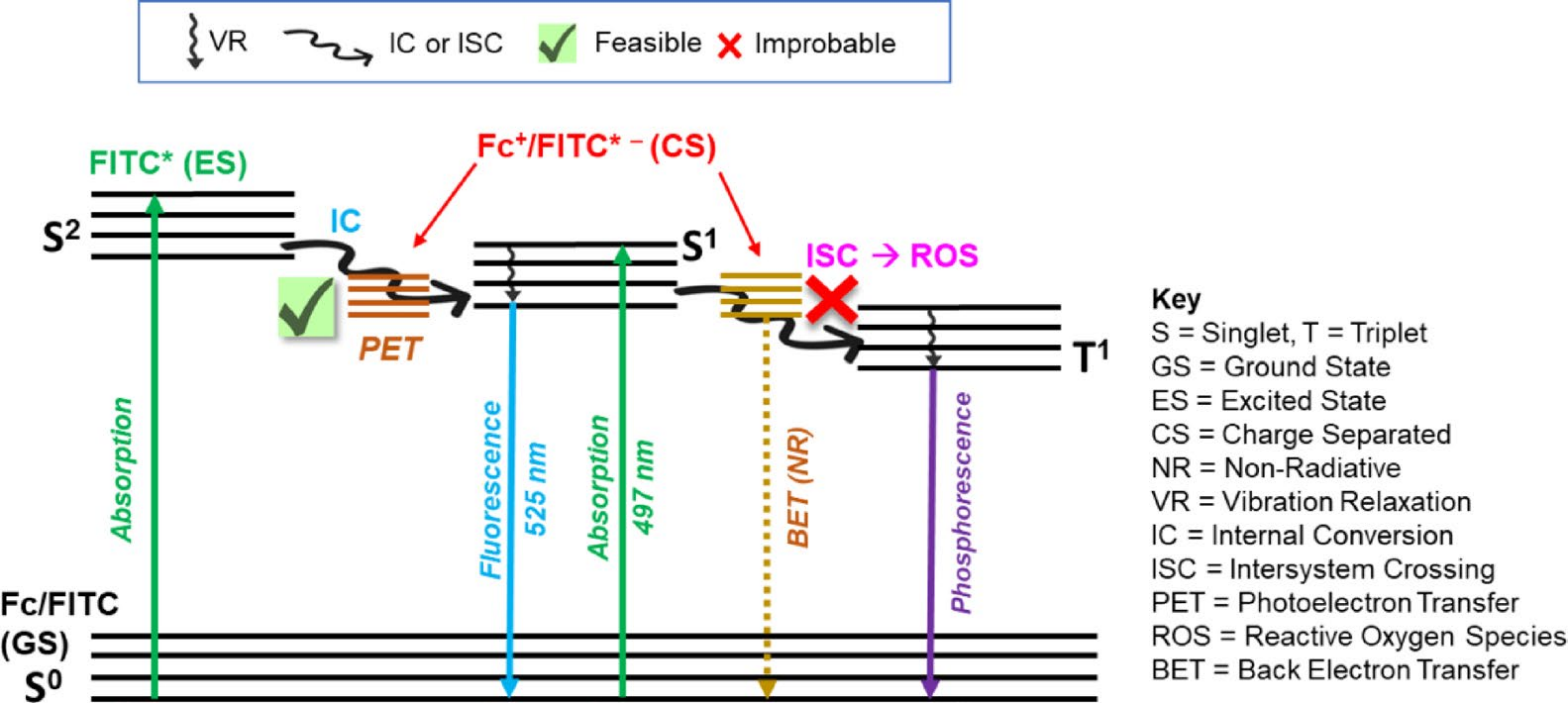
Jablonski diagram of Fc-FITC showing PET to Fc efficiently quenches fluorescence and suppresses ISC to T^1^ with BET providing a protective NR return to the GS.

The entire protective cycle is summarized by the Jablonski diagram (Fig. 8). The energy diagram illustrates how PET outcompetes the destructive ISC pathway. Excitation at 497 nm promotes FITC to the excited state (S^1^​​ or S^2^​). This state typically decays via fluorescence or ISC to the triplet state (T^1^​), which forms ROS and causes photobleaching. In the Fc–FITC conjugate, PET occurs from the HOMO of Fc to the LUMO of FITC*(S^1^​), leading to a charge-separated (CS) state (Fc^+^/FITC*^−^) that outcompetes ISC. The cycle is completed by rapid BET from the CS state back to the singlet ground state (S^0^​), providing a protective non-radiative (NR) decay route. Collectively, these findings establish a robust framework for mitigating fluorophore photodegradation through the covalent incorporation of redox-active ferrocene. Future work will employ both transient absorption spectroscopy to directly observe the sub-nanosecond kinetics of the PET and BET pathways and rotating disk electrode (RDE) measurements for further electrochemical refinement.

### Singlet oxygen validation

To directly validate the suppression of ROS formation, comparative singlet oxygen (^1^O_2_) generation studies were performed using Singlet Oxygen Sensor Green (SOSG) assay^73–75^. Under matched excitation and photon absorption conditions (A_505_ ​= 0.115), the apparent singlet oxygen generation rate for Fc–FITC was found to be only 28% of that for native FITC (1.46 × 10^4^ vs. 4.09 × 10^3^ a.u./min^1^, Figures S40-41. The specificity of the assay was confirmed through the addition of sodium azide (NaN₃, 10 mM), a known ^1^O_2_ quencher^76–78^. For the FITC system, the addition of NaN₃ after 30 min of irradiation led to a 32.5% reduction in fluorescence, confirming that ^1^O_2_ was the primary reactive species driving the SOSG response. In contrast, the Fc–FITC system exhibited only a 4.6% quenching effect, a value nearly identical to the SOSG control (5.1%). These results, summarized in Table S5, confirm that the SOSG fluorescence increase in the FITC system arises predominantly from ^1^O_2_ oxidation, while the Fc–FITC system produces minimal singlet oxygen. These findings collectively demonstrate that redox-active Fc substitution modulates excited-state dynamics, effectively suppressing triplet-state formation and ROS sensitization efficiency through the intramolecular PET pathway.

## Conclusion

This study addresses photobleaching, a major limitation found in fluorescence based applications, by synthesizing a novel ferrocene-fluorescein conjugate (Fc-FITC). We successfully demonstrated that covalently linking the fluorescein core to the redox-active ferrocene moiety significantly enhances photostability through intramolecular excited-state quenching. Comprehensive photophysical and electrochemical characterization confirmed efficient quenching, evidenced by an 81.5% reduction in fluorescence quantum yield and a shortened lifetime (*τ* = 3.2 ns). The Fc-FITC conjugate demonstrated a significant and quantifiable photostability enhancement, showing an 11-fold increase in photobleaching half-life (693 min vs. 63 min for FITC) and retaining 94% of its initial fluorescence after 60 min of continuous illumination. While the conjugate exhibits a lower initial quantum yield compared to native FITC, we establish that for quantitative sensing applications, signal precision (stability) is more critical than signal magnitude (brightness). The 94% retention of Fc-FITC provides the stable baseline required for accurate long-term monitoring, which is unattainable with the rapidly decaying signal of FITC. This enhancement is attributed to a Photoinduced Electron Transfer (PET) mechanism, where the ferrocene unit rapidly dissipates the excited-state energy. The PET is thermodynamically confirmed as highly favorable (ΔG_PET_​ ≈ −0.76 eV) via Rehm–Weller analysis, and the process effectively suppresses the intersystem crossing pathway, minimizing the formation of the destructive triplet state and subsequent reactive oxygen species (ROS). This mechanism was experimentally validated by SOSG assays, which revealed that Fc–FITC produces 72% less singlet oxygen than native FITC under identical irradiation, with sodium azide quenching confirming the specificity of this suppression. Furthermore, computational analysis supports this, showing that the polar solvent environment forces the system into the highly efficient Fc→FITC* PET channel. This work establishes a reliable molecular design strategy for creating robust fluorescent probes with dual optical and electrochemical functionality for applications in persistent sensing and long-term quantitative analysis. For complete mechanistic validation, future studies will focus on transient absorption spectroscopy to directly observe the sub-nanosecond kinetics, rotating disk electrode (RDE) measurements for electron counting (n) to further quantify the efficiency of ROS suppression across varied environments.

## Materials and methods

### Chemicals and instrumentation

All chemicals and reagents were primarily sourced from Sigma Aldrich (MilliporeSigma) and used as received without further purification. Key reagents included ferrocene methanol (FcCH_2_OH), sodium azide (NaN_3_​), glacial acetic acid (CH_3_​COOH), activated zinc powder (Zn), ammonium chloride (NH_4_​Cl), fluorescein isothiocyanate (FITC, isomer I), N, N-dimethylformamide (DMF), triethylamine (TEA), and dichloromethane (DCM). High-Performance Liquid Chromatography (HPLC)-grade solvents were used for purification. Deionized water (DI water) was generated in-house. Detailed descriptions of all analytical instrumentation, including Nuclear Magnetic Resonance (NMR) spectroscopy, High-Resolution Mass Spectrometry (HRMS), Fourier Transform Infrared (FTIR) spectroscopy, UV-Vis absorption, fluorescence spectroscopy, electrochemistry (CV/DPV), and the photostability assessment system, are provided in the SI, Section S1.

### Synthesis of Fc-FITC

The Fc-FITC conjugate was prepared via a two-step functionalization sequence, starting with commercially available ferrocene methanol (FcCH_2_OH). The synthesis of the key intermediate, ferrocene methylene amine (FcCH_2_NH_2_​​), involved the preparation and subsequent zinc-mediated reduction of the azide intermediate (FcCH_2_N_3_​). The full experimental protocol and spectroscopic confirmation data (NMR, HRMS, and FTIR data) for all intermediates are detailed in the SI (Section S2, Figures S1–S22). The final conjugation step was performed by reacting FcCH_2_NH_2_​​ (27.0 mg, 0.126 mmol) with FITC (49 mg, 0.126 mmol). The reactants were dissolved in anhydrous DMF (3.0 mL) under an inert nitrogen atmosphere. Triethylamine (TEA; 20.0 µL, 0.126 mmol) was added as a base, and the mixture was stirred at room temperature for 24 h in the dark to form the stable thiourea linkage (Fig. 1). Purification of the crude product was achieved using flash column chromatography (CombiFlash NEXTGEN 300+, 40 g silica column) with a DCM − MeOH solvent gradient (Figure S3). The isolated yield was ∼55%. Spectroscopic confirmation for the final conjugate is available in the SI (Figures S23–S25). ^1^H NMR (300 MHz, DMSO-d_6_): δ 4.05–4.10 (br, 2 H, –CpCH_2_N), 4.2 (s, 5 H, Cp-H), 4.3 (s, 2 H, Cp-H), 4.5 (s, 2 H, Cp-H, 6.5–6.75 (br, 6 H, –FITC), 7.2 (d, 1H, –FITC), 7.75 (d, 1H, –FITC), 8.1 (s, 1H, –CH_2_NH-C = S), 8.3 (s, 1H, –FITC), 9.9 (s, 1H, –CNH-C = S), 10.2 (d, 2 H, –FITC). HRMS of Fc-FTIC: {(C_32_H_24_FeN_2_O_5_S less H^–^} calculated m/z = 603.0678, z = − 1, found 603.0716, Δ ppm = 6.30.

### Spectroscopy and electrochemistry

Details regarding the preparation of all pH buffers (Table S1), the specific conditions for p*K*a​ determination, fluorescence quantum yield (Φ_F_​) calculation, and Stern–Volmer analysis are provided in the SI (Sections S5 and S6). Cyclic voltammetry (CV) experiments used a scan range of − 0.5 V to + 1.0 V vs. Ag/Ag^+^ at 100 mV/s. The CV setup was optimized for accurate E_ox_ potential determination under identical comparative conditions, rather than for the precise measurement of kinetic reversibility or the number of electrons (n).

### Computational

All computational studies were performed using the Gaussian 16 software. Calculations utilized Time-Dependent Density Functional Theory (TDDFT) on the deprotonated FITC and Fc-FITC geometries, exploring B3LYP and CAM-B3LYP functionals with the SMD implicit solvation model to mimic the EtOH/H_2​_O experimental environment. Detailed methodology and DFT output figures are available in the SI (Section S7).

## Supplementary Information

Below is the link to the electronic supplementary material.

Supplementary Material 1

## Data Availability

All data generated or analyzed during this study are included in this published article (and its Supplementary Information files). Raw spectral data (NMR, HRMS, FTIR), electrochemical files, and full computational outputs (Gaussian logs) are available from the corresponding authors.
