# Supplementary material for: Covalent ferrocene conjugation as an intramolecular strategy for photostability in fluorescein
Source: Sci Rep. 2026 Jan 7;16:4816. doi: 10.1038/s41598-025-34817-3 (PMC12873245; doi:10.1038/s41598-025-34817-3)
Supplement: Supplementary file 1 — Supplementary Material 1 [file 41598_2025_34817_MOESM1_ESM.pdf]

Supporting Information

Covalent ferrocene conjugation as an intramolecular strategy for photostability in fluorescein

Gilbert K. Kosgei [a, §*]; P.U. Ashvin Iresh Fernando [b, c, §*]; Harley R. Mcalexander [a]; Afrachanna D. Butler [a]

[a] US Army Engineer Research and Development Center, Environmental Laboratory, 3909 Halls Ferry Road, Vicksburg, MS, 39180, USA
[b] US Army Engineer Research and Development Center, Cold Regions Research and Engineering Laboratory, 72 Lyme Road, Hanover, NH 03755, USA
[c] SIMETRI, Inc, Inc. 937 S Semoran Blvd Suite 100, Winter Park, FL 32792, USA

[§]These authors contributed equally to this work
**Corresponding Authors: Gilbert.K.Kosgei@usace.army.mil and Ashvin.I.Fernando@usace.army.mil*

Contents

S1. Materials and instrumentation 2
S2. Synthesis procedures 3
S3. Flash chromatography 4
S4. Compound characterization 5
S5. Buffer solutions 17
S6. Photophysical characterization 18
S7. Computational studies 24
S8. Photostability studies 28
S9. Singlet oxygen ($^1O_2$) generation assay 29

S1. Materials and instrumentation

Materials and reagents

All chemicals and reagents were primarily sourced from Sigma Aldrich (MilliporeSigma) and used as received without further purification. Key reagents included ferrocene methanol ($FcCH_2OH$) (97%), sodium azide (NaN_3) (≥99%), glacial acetic acid (CH_3COOH) (≥99%), Singlet Oxygen Sensor Green (SOSG, Invitrogen), activated zinc powder (Zn) (≤10 µm, ≥98%), ammonium chloride (NH_4Cl) (≥99.5%), fluorescein isothiocyanate (FITC, isomer I) (≥97.5%), N,N-dimethylformamide (DMF) (anhydrous, 99.8%), triethylamine (TEA, Et_3N) (≥99.5%), and dichloromethane (DCM) (anhydrous, ≥99.8%). HPLC-grade chromatography solvents from Fisher Scientific which includes methanol (≥99.9%), ethyl acetate (EtOAc), hexane, acetonitrile (ACN), and absolute ethanol (EtOH) (200 proof). Deionized water (DI water) (≥18.2 MΩ cm^{-1}) was generated in-house using a Milli-Q Advantage system. pH measurements were conducted using a Mettler Toledo SevenCompact Duo meter, and buffers (pH 2–13) were prepared in-house (Table S1).

Instrumentation

Spectroscopy and photophysics: *NMR Spectroscopy*: All ^1H and ^{13}C NMR studies were performed on a Bruker Avance 300 MHz spectrometer. *Mass spectrometry (HRMS):* Spectra were acquired on a Thermo Scientific Q Exactive Orbitrap mass spectrometer using Heated Electrospray Ionization (HESI) in both positive and negative ion modes (180–900 m/z). *FTIR spectroscopy:* The Nicolet iS50 spectrometer (Thermo Fisher Scientific) equipped with a diamond ATR accessory and an MCT detector was used to obtain FTIR-ATR spectra. *UV-Vis absorption*: The Shimadzu UV-2450 spectrophotometer was used to obtain UV-Vis spectra (200 to 800 nm). *Fluorescence spectroscopy*: The FLS 1000 Photoluminescence Spectrometer (Edinburgh Instruments) was used to perform measurements, including photoluminescence excitation/emission and lifetime determination. The excitation source was a 450 W ozone-free xenon arc lamp. The fluorescence decay was measured using a 247 nm diode laser and fitted to a single exponential decay function. All UV-Vis and fluorescence measurements were performed at a working concentration of approximately 2 µM to ensure that absorbance remained below 0.2 A.U. at the excitation wavelength (490–500 nm). This minimized inner-filter effects and maintained spectral accuracy during emission comparisons.

Electrochemistry*:* A PalmSens Multiplexer potentiostat was used to conduct electrochemical measurements (CV and DPV). A standard three-electrode system was used, comprising a glassy carbon working electrode (GCE), a platinum wire (Pt) counter electrode, and an Ag/Ag+ non-aqueous reference electrode. Measurements were conducted in anhydrous acetonitrile (MeCN) containing 0.1 M tetrabutylammonium hexafluorophosphate ($TBAPF_6$) as the supporting electrolyte. CV experiments used a scan range of −0.5 V to +1.0 V vs Ag/Ag+ at 100 mV/s.

Photostability assessment: Continuous irradiation was performed using an OmniCure S1500 Curing System (Excelitas Technologies), featuring a 200-watt mercury vapor short arc lamp. The samples were irradiated at ~320–500 nm (UV-Vis) with a power density of 23 mW/cm². The change in emission intensity at 530 nm was monitored over 60 minutes using the FLS 1000 Photoluminescence Spectrometer. Photobleaching measurements were conducted in a standard cuvette with continuous magnetic stirring to maintain sample homogeneity. The excitation beam uniformly irradiated the full optical volume, eliminating spatial gradients and ensuring reproducible decay kinetics.

S2. Synthesis procedures

The ferrocene methylene amine ($FcCH_2NH_2$) intermediate was prepared via a two-step functionalization of ferrocene methanol ($FcCH_2OH$) involving an azide substitution followed by a zinc-mediated reduction.

Ferrocene methylene azide ($FcCH_2N_3$) synthesis

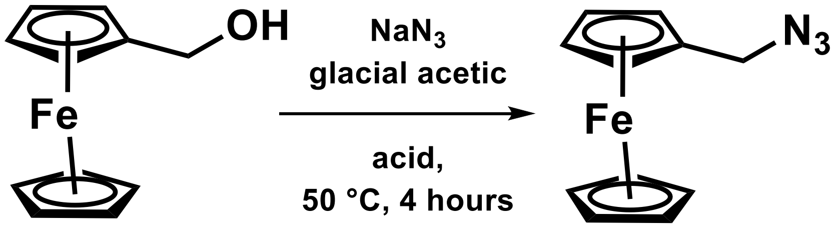

Figure S1: $FcCH_2N_3$ synthesis from $FcCH_2OH$.

Ferrocene methanol ($FcCH_2OH$; 133 mg, 0.60 mmol) and sodium azide (NaN_3; 240 mg, 3.69 mmol) were dissolved in glacial acetic acid (3.0 mL) under an inert atmosphere. The reaction vessel was purged with nitrogen for 15 minutes to remove residual oxygen. The mixture was stirred at 50°C for 4 hours. (Figure S1). After the reaction, the mixture was diluted with DCM, and the organic phase was washed (3×50 mL) with saturated aqueous sodium bicarbonate solution ($NaHCO_3$) to neutralize the excess acid. The organic layer was separated, dried over anhydrous sodium sulfate, filtered, and concentrated via rotary evaporation to yield a crude brown/orange oil. The product solidified into a yellowish-brown solid under vacuum. ^{1}H NMR (300 MHz, DMSO-d_6): δ 4.19 – 4.23 (br, 9H, Cp-H), 4.29 – 4.32 (br, 2H, CH_2N_3). ^{13}C NMR (75 MHz, DMSO-d_6): δ 50.10 (CH_2N_3), 68.50, 68.60, 68.70 (Cp carbons), 82.10 (quaternary Cp-C). FTIR: A sharp peak at 2092 cm^{-1} confirmed the presence of the characteristic N_3 stretch. (HRMS acquisition was challenging due to azide group fragmentation.)

Ferrocene methylene amine ($FcCH_2NH_2$) synthesis

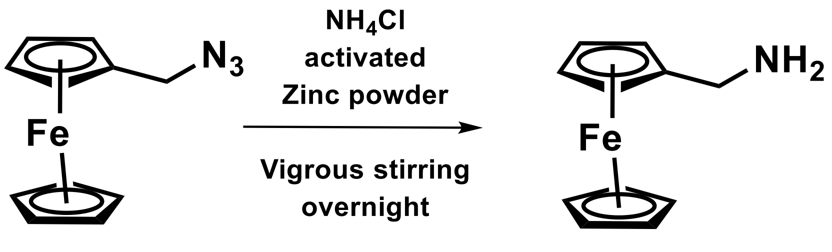

Figure S2. The reduction of $FcCH_2N_3$ to $FcCH_2NH_2$

Ferrocene methylene azide ($FcCH_2N_3$) was dissolved in a mixture of ethanol and deionized water (9:1 v/v). Ammonium chloride (NH_4Cl) was added as a proton source, followed by the introduction of activated zinc powder. The suspension was vortexed vigorously (20–30 minutes) and then stirred overnight (~600 rpm) at room temperature to ensure complete reduction (Figure S2).

The reaction mixture was subjected to vacuum filtration to remove excess zinc and byproducts. The solid residue was thoroughly washed with ethyl acetate (EtOAc). The combined filtrate was concentrated, redissolved in EtOAc (40 mL), and extracted with 1 N aqueous ammonia (30 mL) to basify the amine product. The aqueous layer was separated and re-extracted with EtOAc (2×40 mL). All organic phases were combined, dried over anhydrous sodium sulfate, filtered, and concentrated under vacuum. The crude product, a slightly dark yellow residue, was subjected to high vacuum drying for 24 hours prior to use. Obtained yield of ~50 to 60%. ^{1}H NMR (300 MHz, DMSO-d_6): δ 3.41 (s, 2H, NH_2), 4.05 – 4.10 (br, 2H, CH_2NH_2), 4.11 – 4.25 (br, 9H, Cp-H). ^{13}C NMR (300 MHz, DMSO-d_6): δ 50.1 (CH_2NH_2), 67.0, 68.0, 68.3 (Cp carbons), 88.1 (quaternary Cp-C) HRMS of $FcCH_2NH_2$: {($C_{11}H_{11}$Fe less NH_2^-} calculated m/z = 199.0210, found 199.0202, Δ ppm = 4.01, where z = 1.

S3. Flash chromatography

Flash chromatography was performed using a CombiFlash NEXTGEN 300+ system (Teledyne ISCO) fitted with a 40 g silica column for the final purification of the Fc-FITC conjugate. The separation utilized a gradient solvent system of dichloromethane (DCM) and methanol (MeOH) at a flow rate of 60 mL/min. The column was equilibrated with 275 mL of the initial solvent mixture before loading. The crude material was applied in solid form using the system's *pause mode* to ensure precise column loading. Detection relied on a photodiode array detector (PDA), set primarily to 254 nm and 285 nm (peak width: 2 min, threshold: 0.20 AU), with monitoring over the 200–700 nm wavelength range. Both peak and non-peak tube volumes were set to the maximum to ensure thorough fraction collection.

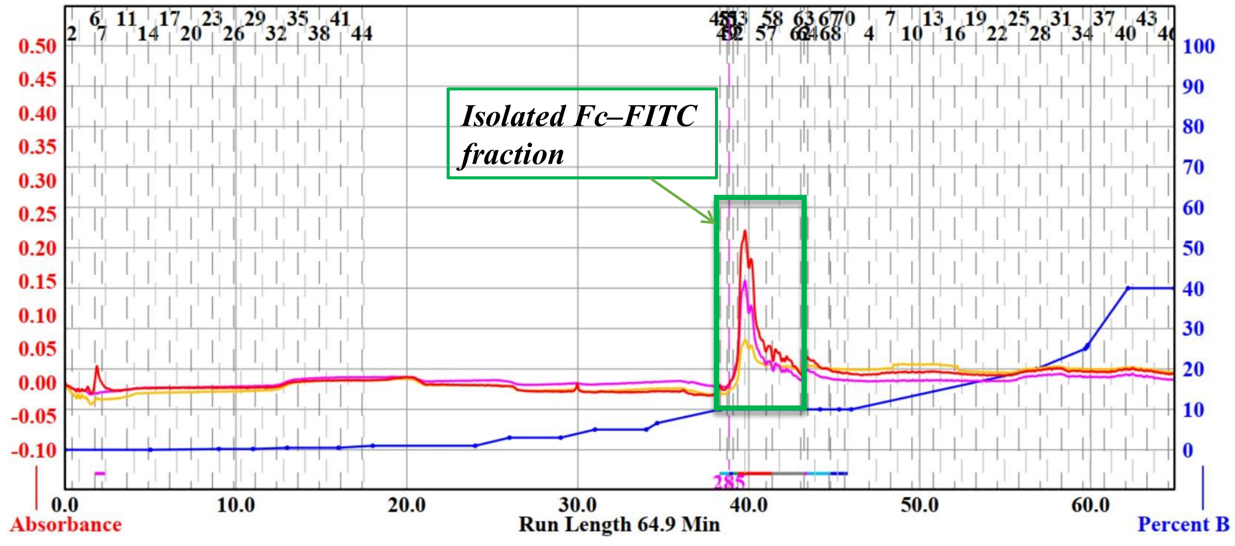

Figure S3: Flash chromatography purification of Fc–FITC. The chromatogram shows the elution profile (DCM–MeOH gradient), with the highlighted peak indicating the collected product fraction.

S4. Compound characterization

(A) Ferrocene methanol ($FcCH_2OH$) – For reference purposes

(i) ^{1}H NMR reference spectrum of ferrocene methanol ($FcCH_2OH$)

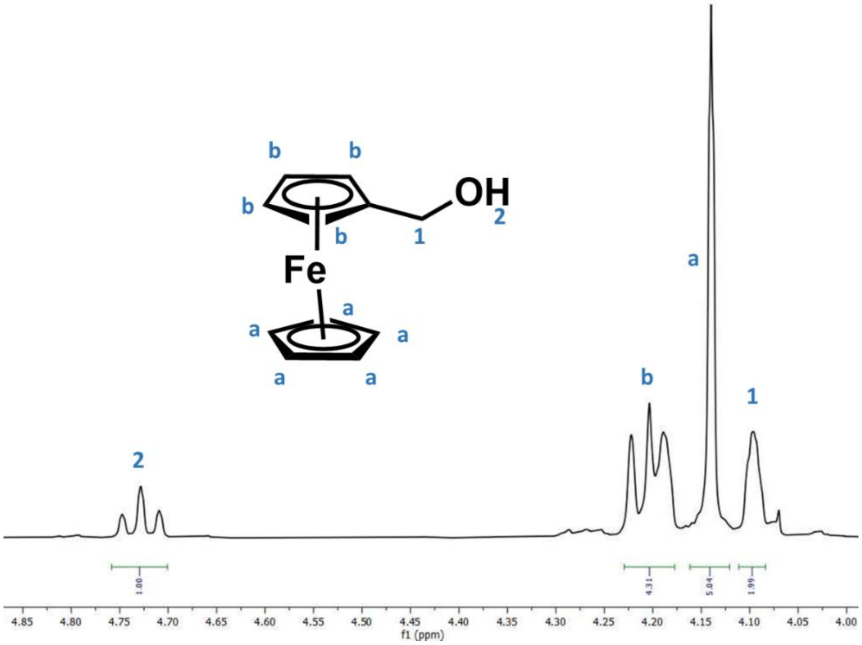

Figure S4: ^{1}H NMR zoomed spectrum of ferrocene methanol ($FcCH_2OH$)

^{1}H NMR (300 MHz, DMSO-d_6): δ4.10 (s, 2H, CH_2OH), 4.14 (s, 5H, Cp-H), 4.20 (t, 4H, Cp-H), 4.73 (t, 1H, OH).

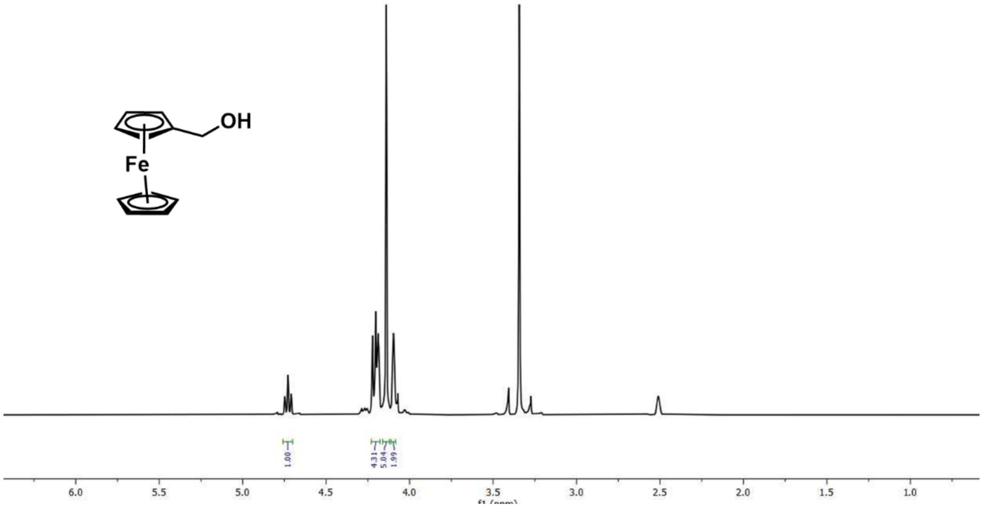

Figure S5: ^{1}H NMR full spectrum of ferrocene methanol ($FcCH_2OH$)

(ii) ^{13}C NMR reference spectrum of ferrocene methanol ($FcCH_2OH$)

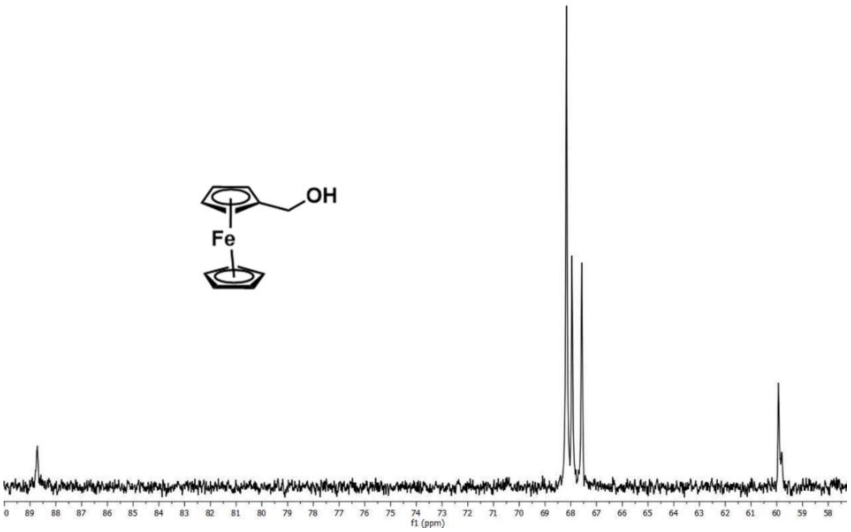

Figure S6: ^{13} C NMR spectrum of ferrocene methanol ($FcCH_2OH$) ^{13}C NMR (300 MHz, DMSO-d_6): δ 59.7 (CH_2OH), 67.5, 68.1, 68.2 (Cp carbons), 88.6 (quaternary Cp-C)

(iii) HRMS of ferrocene methanol ($FcCH_2OH$)

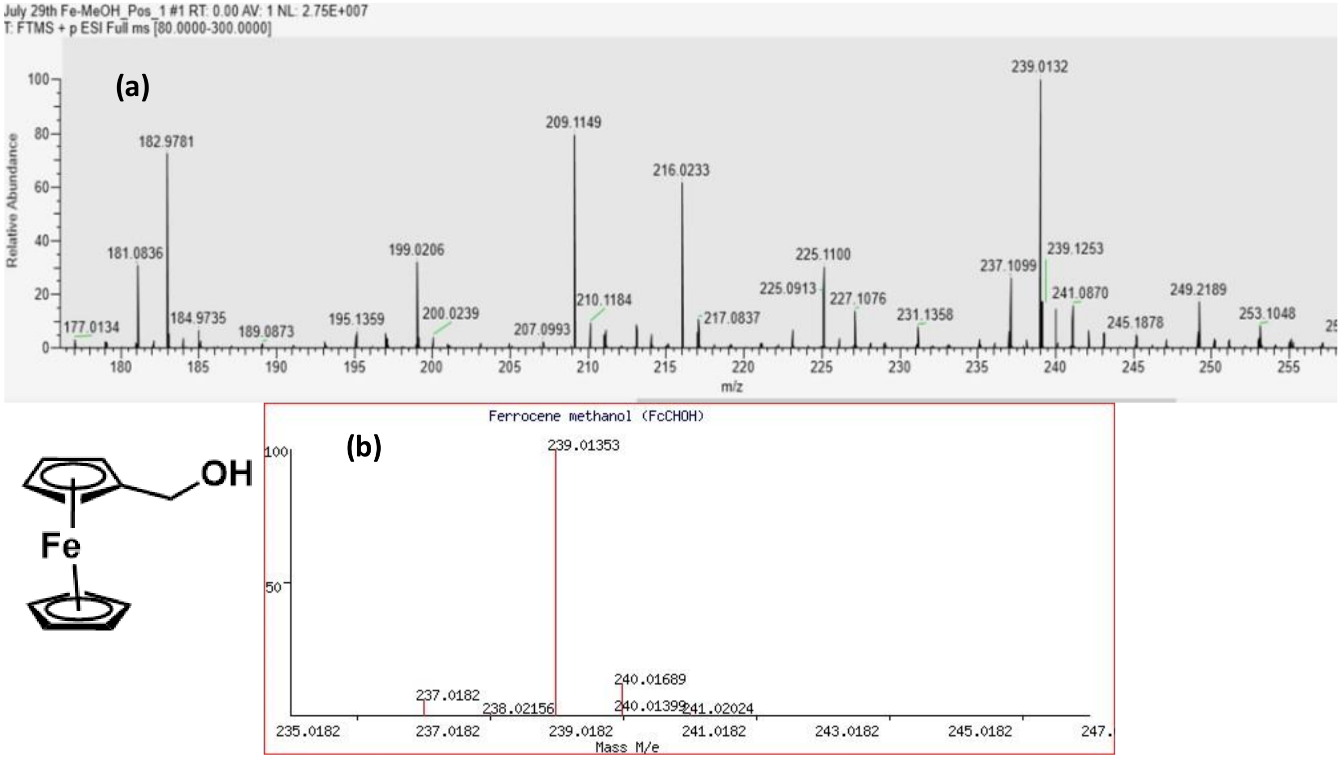

Figure S7: Mass spectrum of $FcCH_2OH$: (a) experimental spectrum; (b) simulated spectrum of the sodium adduct $[FcCH_2OH + Na]^+$. The simulated spectrum reflects the experimentally observed abundant mass and was generated using an online tool. https://www.sisweb.com/mstools/isotope.html $FcCH_2OH$: {($C_{11}H_{12}FeO$ add Na^+} calculated m/z = 239.0135, found 239.0132, Δ ppm = 1.25, where z = 1.

(iv) FTIR spectra of ferrocene methanol ($FcCH_2OH$)

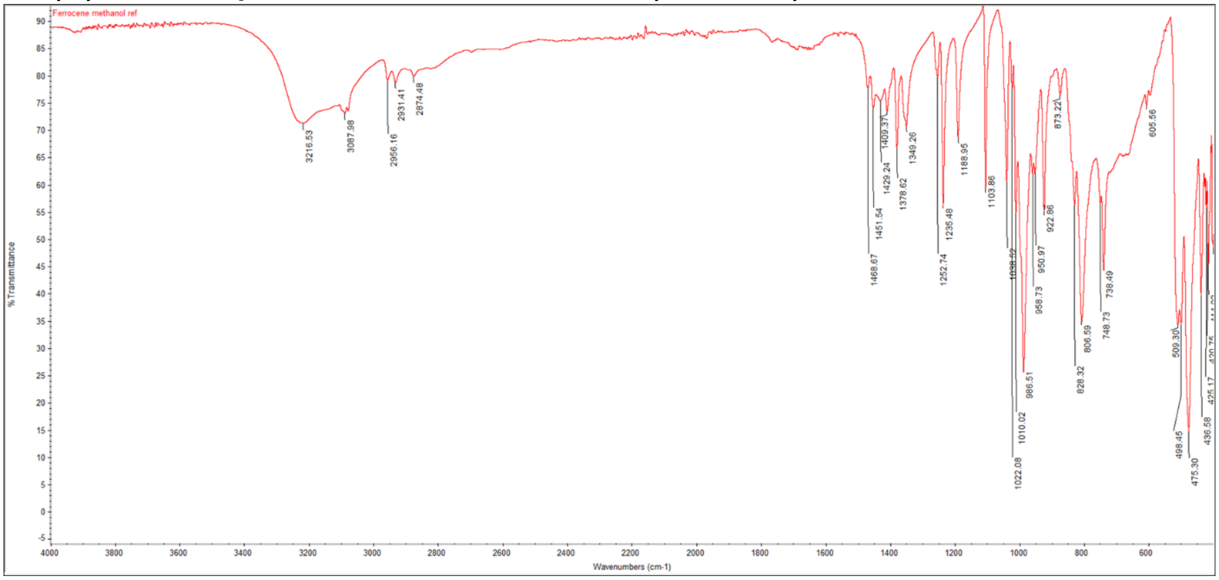

Figure S8: FTIR spectrum of ferrocene methanol ($FcCH_2OH$)

(B) Ferrocene methylene azide ($FcCH_2N_3$)

(i) ^{1}H NMR spectrum of ferrocene methylene azide ($FcCH_2N_3$)

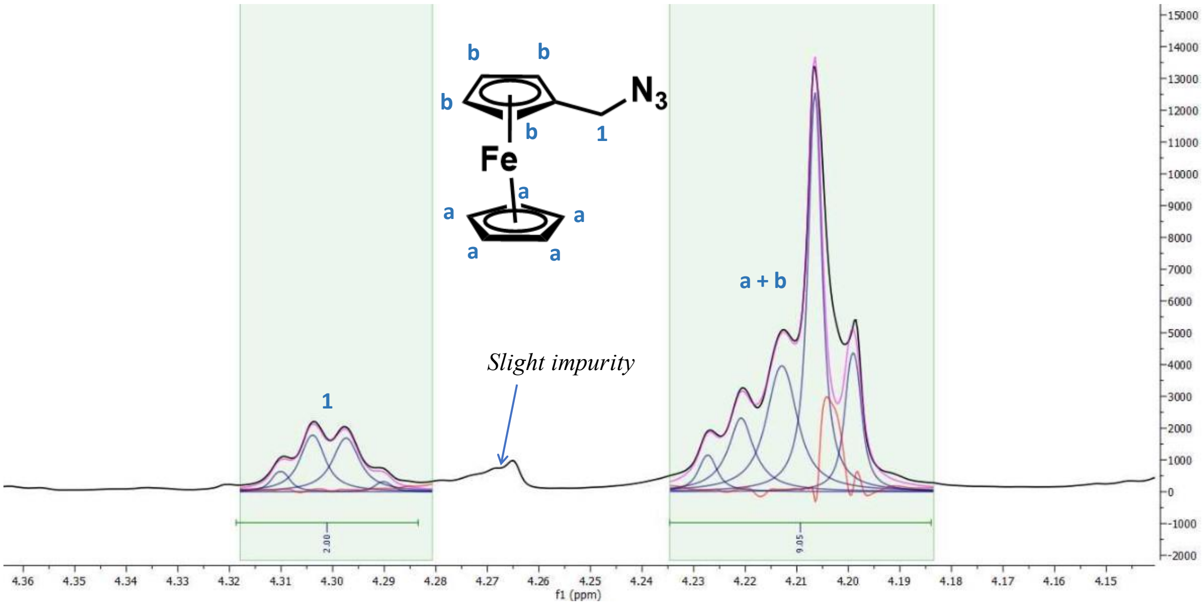

Figure S9: ^{1}H NMR zoomed and fitted spectrum of ferrocene methylene azide ($FcCH_2N_3$)

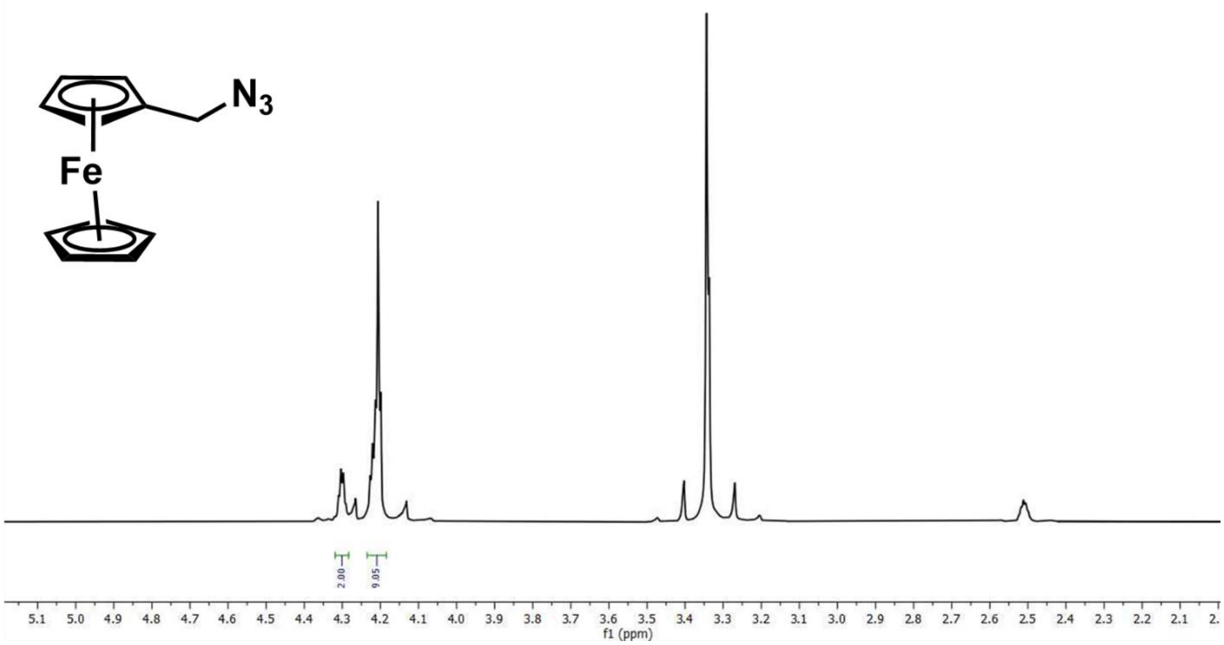

Figure S10: ^{1}H NMR full spectrum of ferrocene methylene azide ($FcCH_2N_3$)

(ii) ^{13}C NMR spectrum of ferrocene methylene azide ($FcCH_2N_3$)

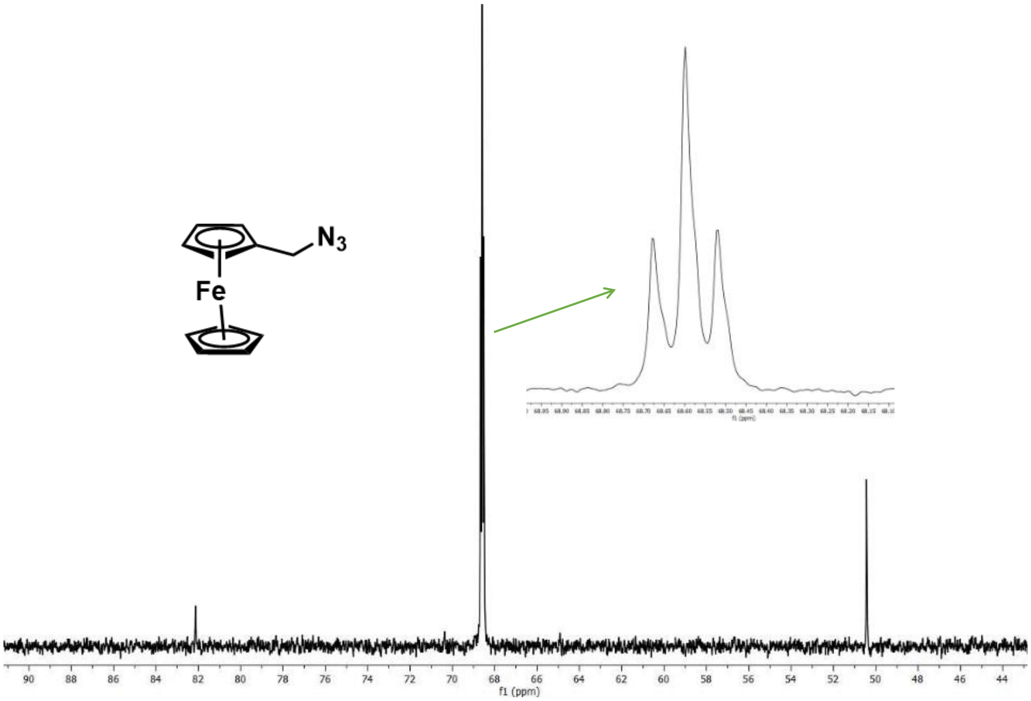

Figure S11: ^{13}C NMR full spectrum of ferrocene methylene azide ($FcCH_2N_3$)

(iii) HRMS of ferrocene methylene azide ($FcCH_2N_3$)

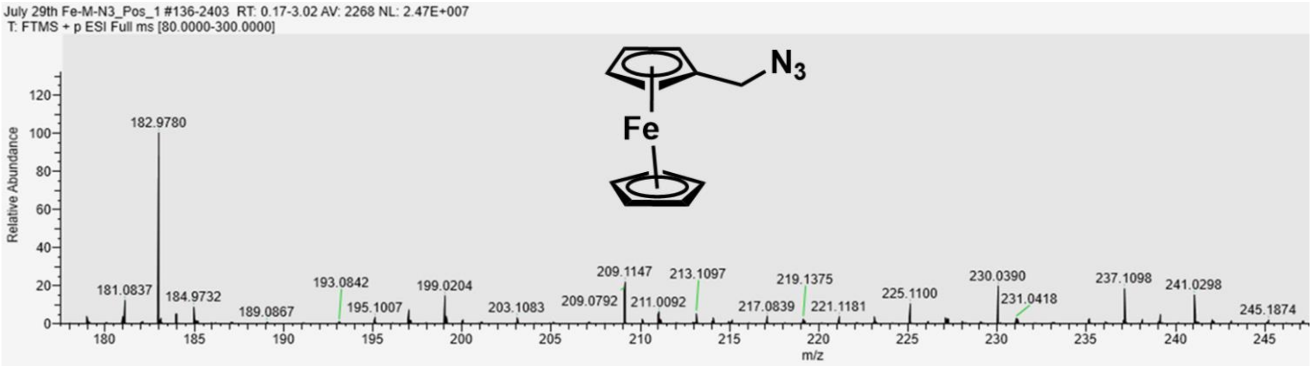

Figure S12: Mass spectrum of ferrocene methylene azide ($FcCH_2N_3$) obtained.

(iv) FTIR spectrum of ferrocene methylene azide ($FcCH_2N_3$)

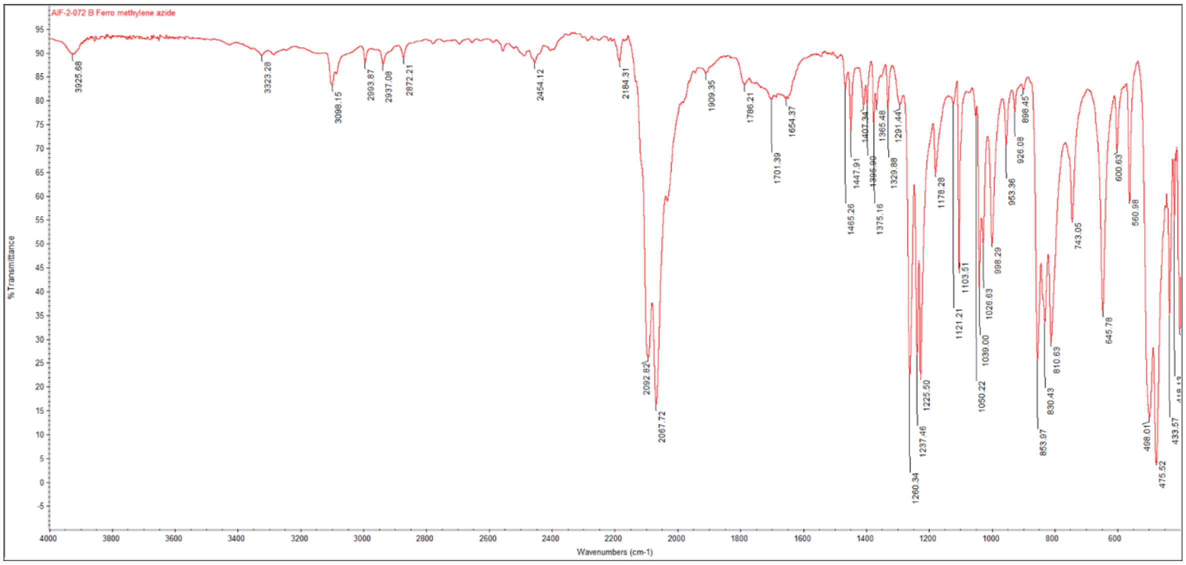

Figure S13: FTIR spectrum of ferrocene methylene azide ($FcCH_2N_3$)

(C) **Ferrocene methylene amine ($FcCH_2NH_2$)**

(i) ^{1}H NMR spectrum of ferrocene methylene amine ($FcCH_2NH_2$)

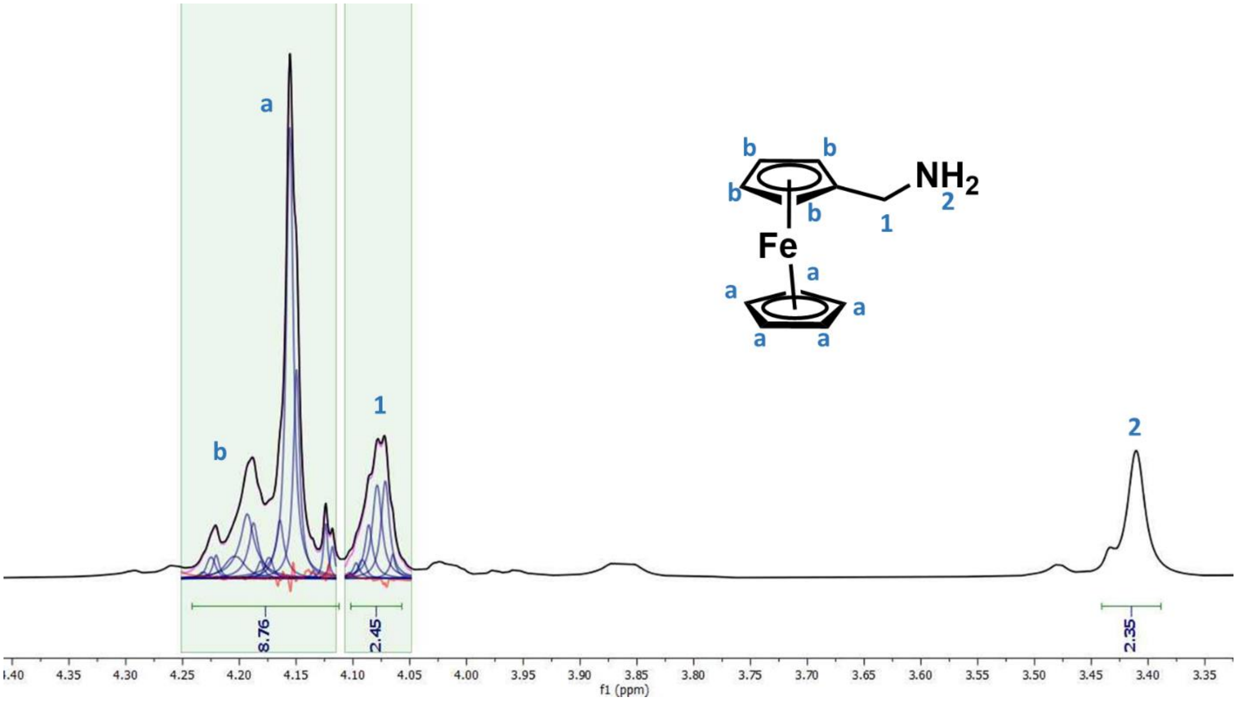

Figure S14: ^{1}H NMR zoomed and fitted spectrum of ferrocene methylene amine ($FcCH_2NH_2$)

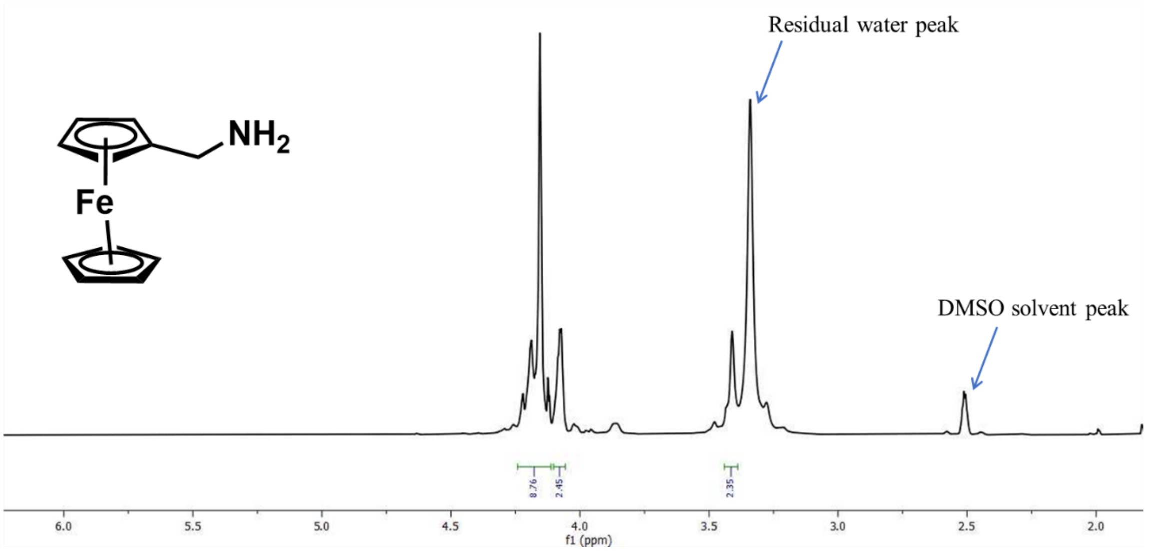

Figure S15: ^{1}H NMR full spectrum of ferrocene methylene amine ($FcCH_2NH_2$)

(ii) ^{13}C NMR spectrum of ferrocene methylene amine ($FcCH_2NH_2$)

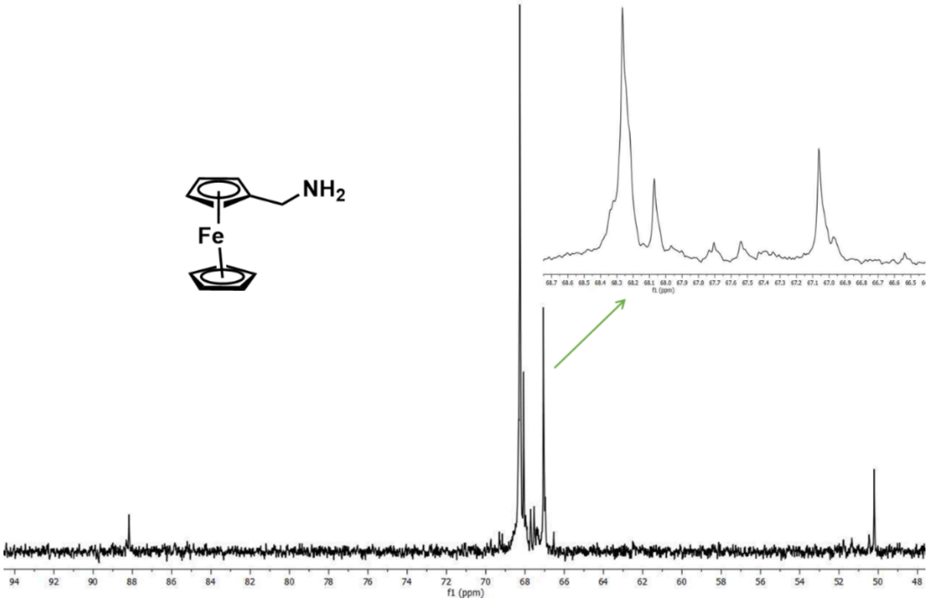

Figure S16: ^{13}C NMR spectrum of ferrocene methylene amine ($FcCH_2NH_2$)

(iii) HRMS of ferrocene methylene amine ($FcCH_2NH_2$)

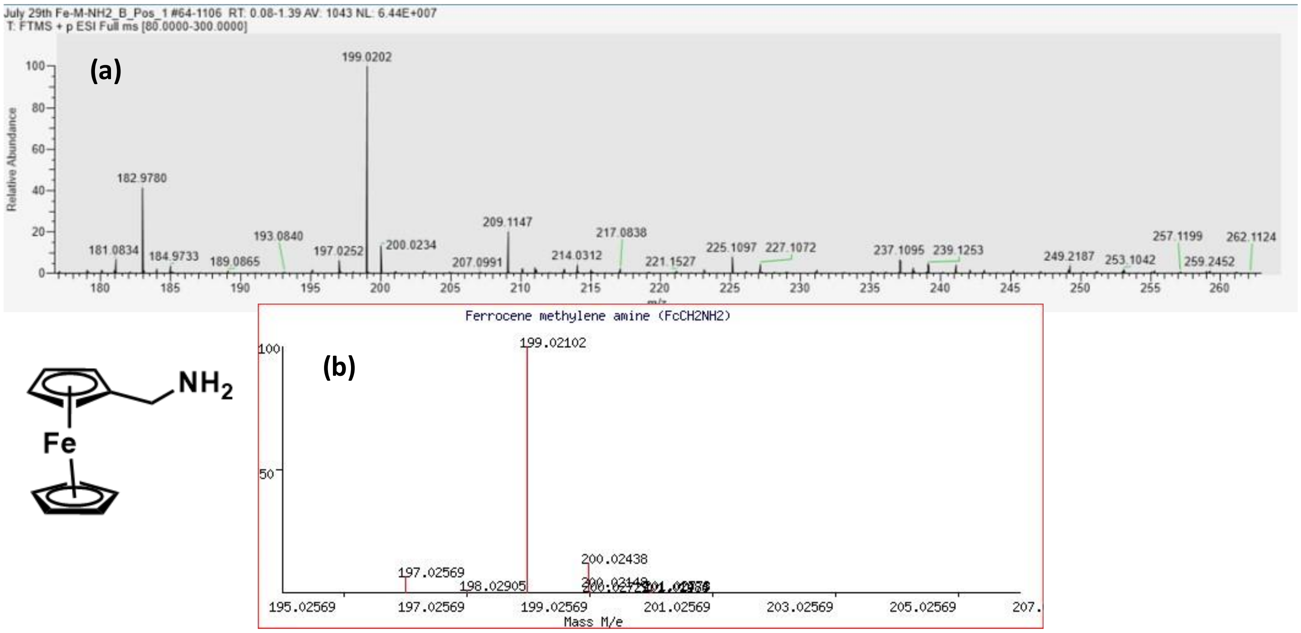

Figure S17: Mass spectrum of $FcCH_2NH_2$: (a) experimental spectrum; (b) simulated spectrum of cleaved amine [$FcCH_2$ – NH_2]. The simulated spectrum reflects the experimentally observed abundant mass and was generated using an online tool: https://www.sisweb.com/mstools/isotope.html

(iv) FTIR spectrum of ferrocene methylene amine ($FcCH_2NH_2$)

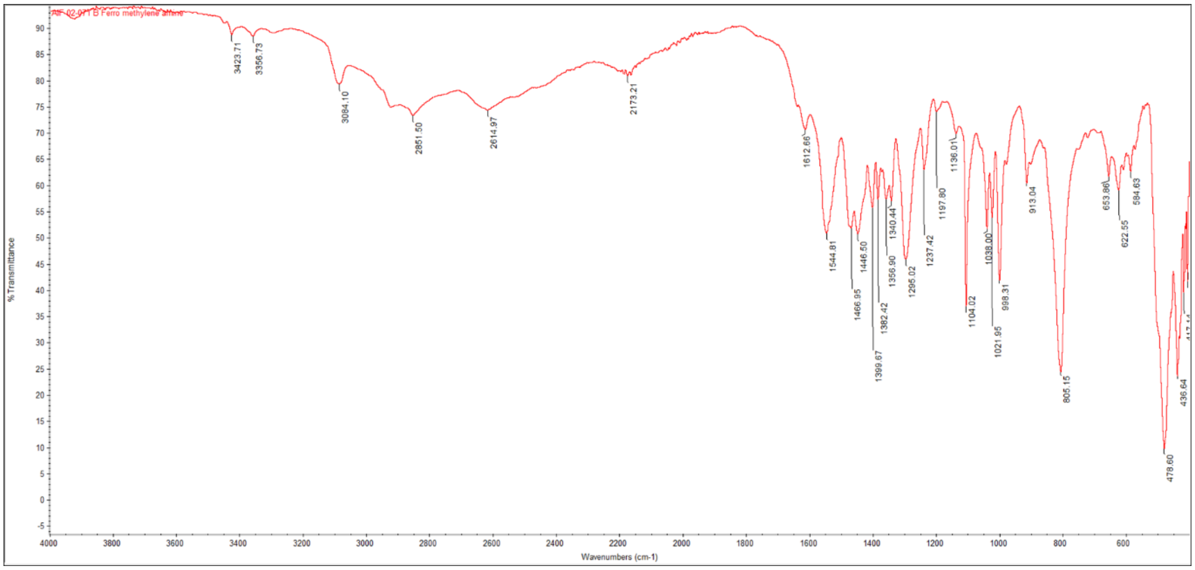

Figure S18: FTIR spectrum of ferrocene methylene amine ($FcCH_2NH_2$)

(D) Stacked spectra of $FcCH_2OH$, $FcCH_2N_3$, and $FcCH_2NH_2$

(i) Stacked ^1H NMR spectra of $FcCH_2OH$, $FcCH_2N_3$, and $FcCH_2NH_2$

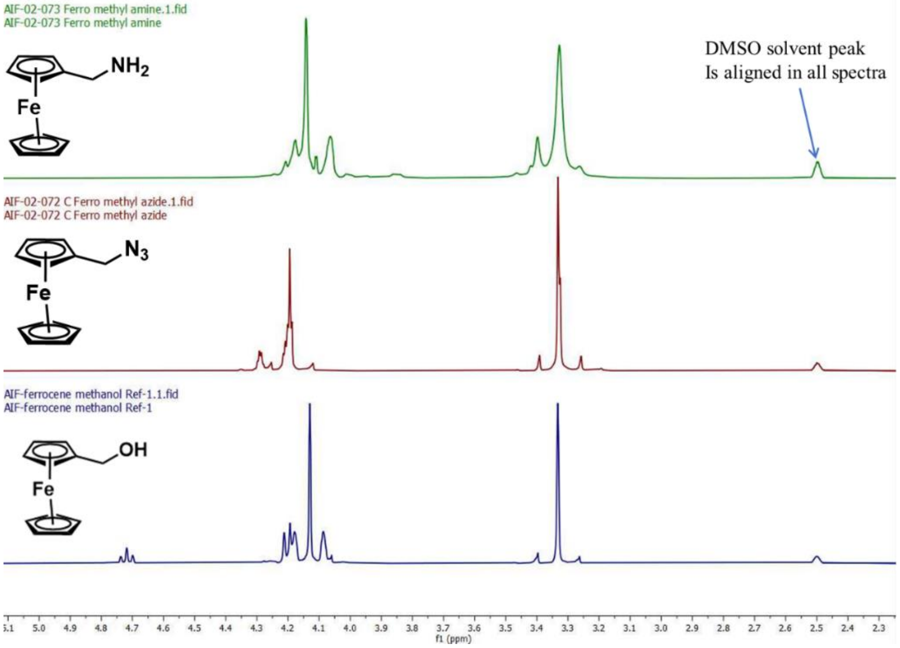

Figure S19: Stacked ^1H NMR spectra of $FcCH_2OH$, $FcCH_2N_3$, and $FcCH_2NH_2$, showing stepwise chemical shift and signal changes across the synthetic sequence

(ii) Stacked ^{13}C NMR spectra of $FcCH_2OH$, $FcCH_2N_3$, and $FcCH_2NH_2$

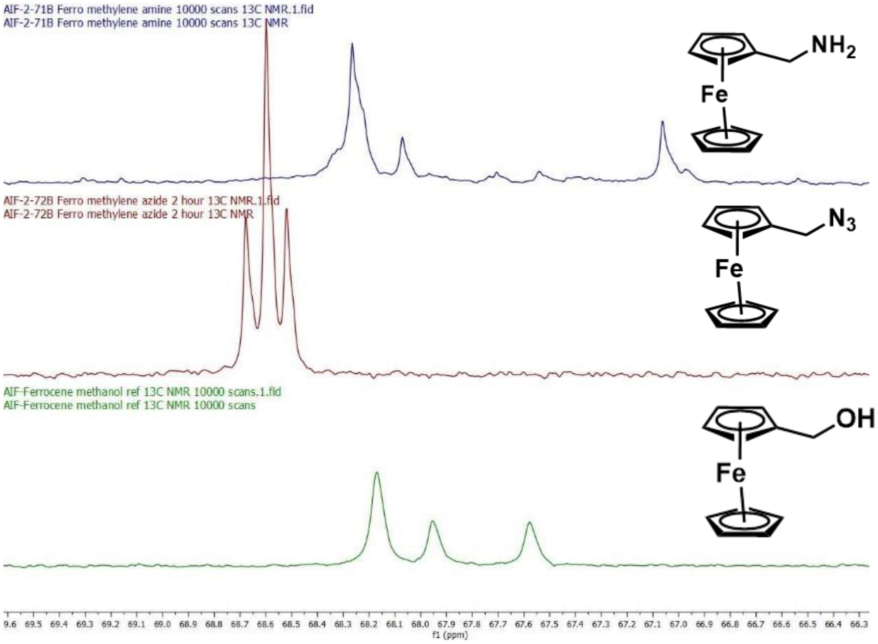

Figure S20: Stacked zoomed ^{13}C NMR spectra of $FcCH_2OH$, $FcCH_2N_3$, and $FcCH_2NH_2$, showing stepwise chemical shift and signal changes across the synthetic sequence

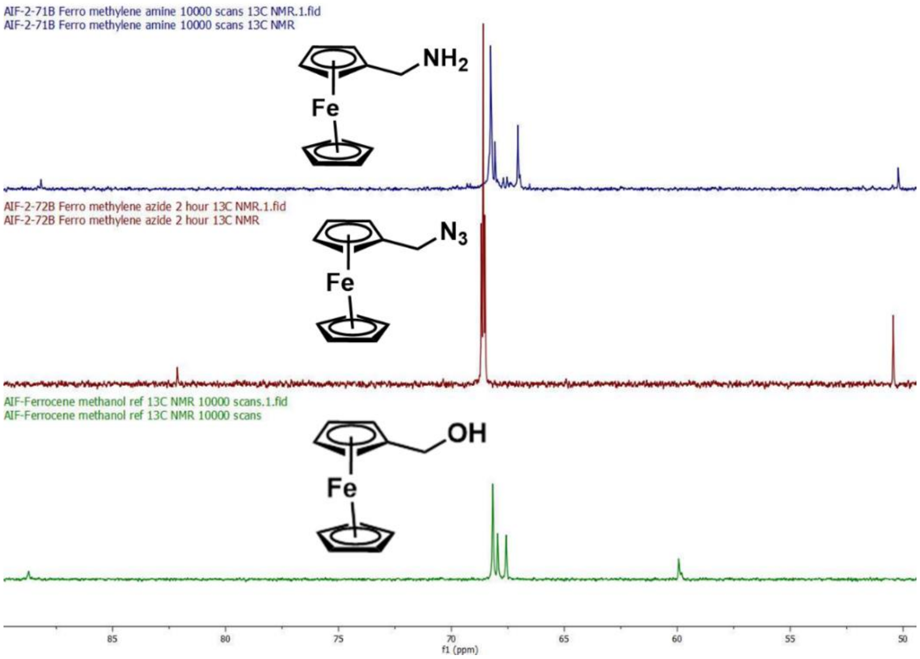

Figure S21: Stacked full ^{13}C NMR spectra of $FcCH_2OH$, $FcCH_2N_3$, and $FcCH_2NH_2$

(iii) Stacked FTIR spectra of $FcCH_2OH$, $FcCH_2N_3$, and $FcCH_2NH_2$

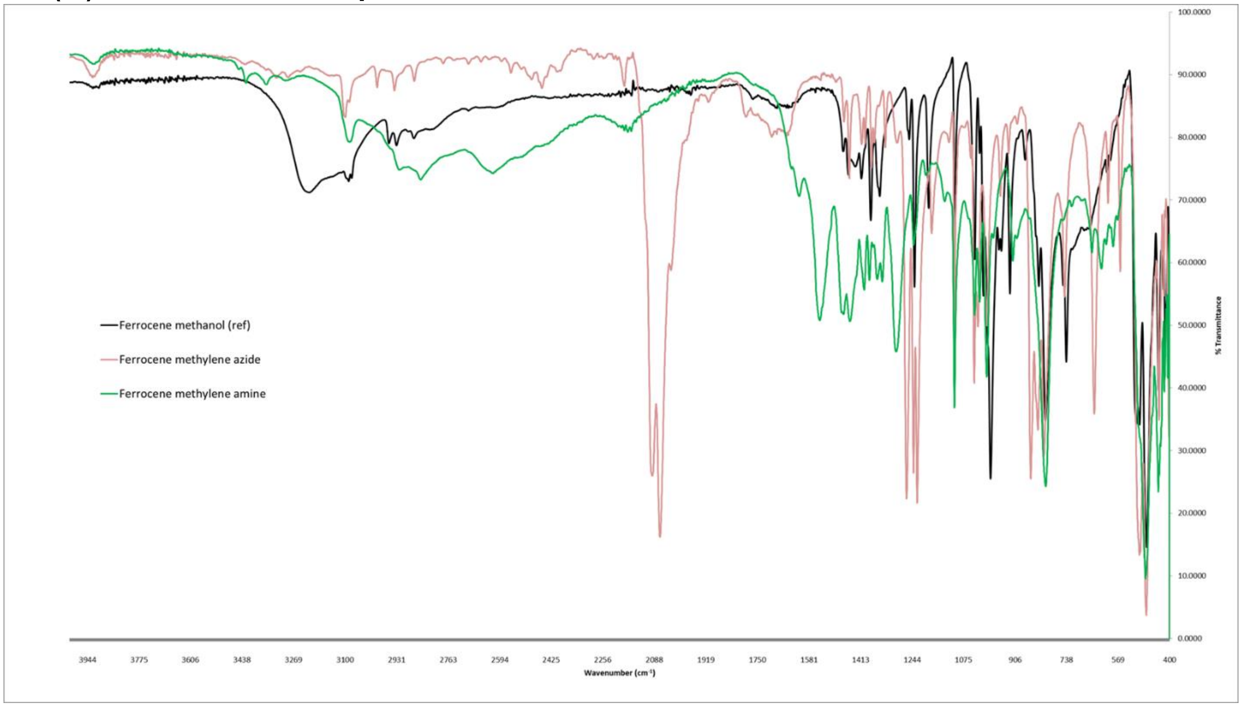

Figure S22: Stacked FTIR spectra of $FcCH_2OH$ (black), $FcCH_2N_3$ (red), and $FcCH_2NH_2$ (green)

(E) Ferrocene-fluorescein conjugate (Fc-FITC)

(i) ^{1}H NMR spectrum of ferrocene-fluorescein conjugate (Fc-FITC)

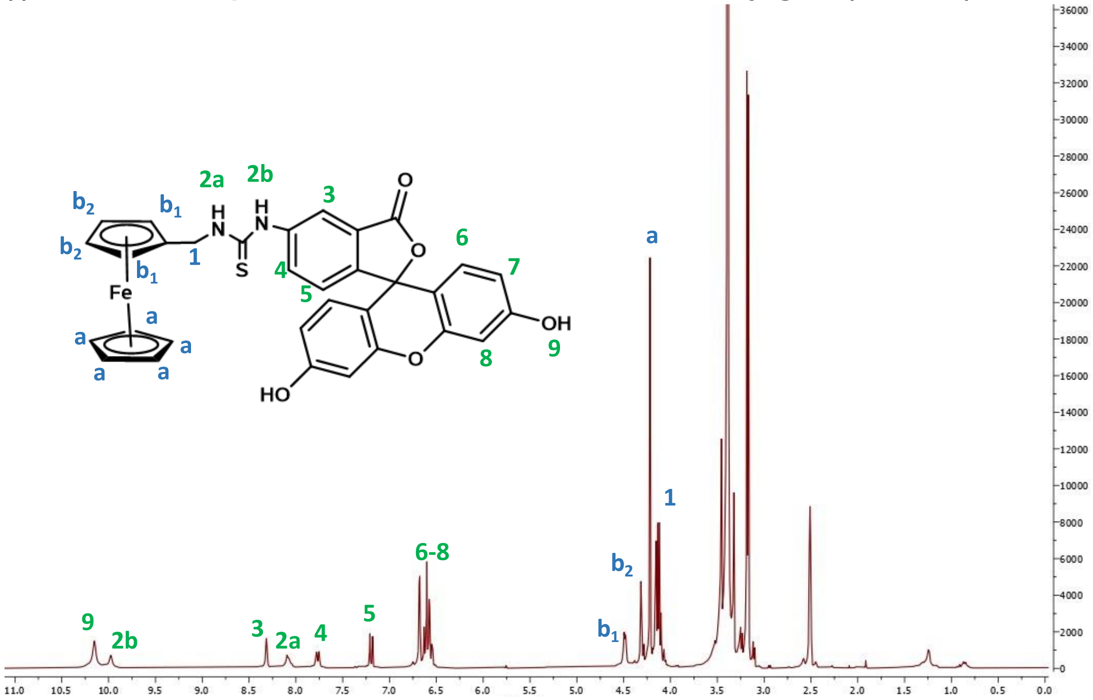

Figure S23: ^{1}H NMR spectrum of ferrocene-fluorescein conjugate (Fc-FITC)

(ii) ^{13}C NMR spectrum of ferrocene-fluorescein conjugate (Fc-FITC)

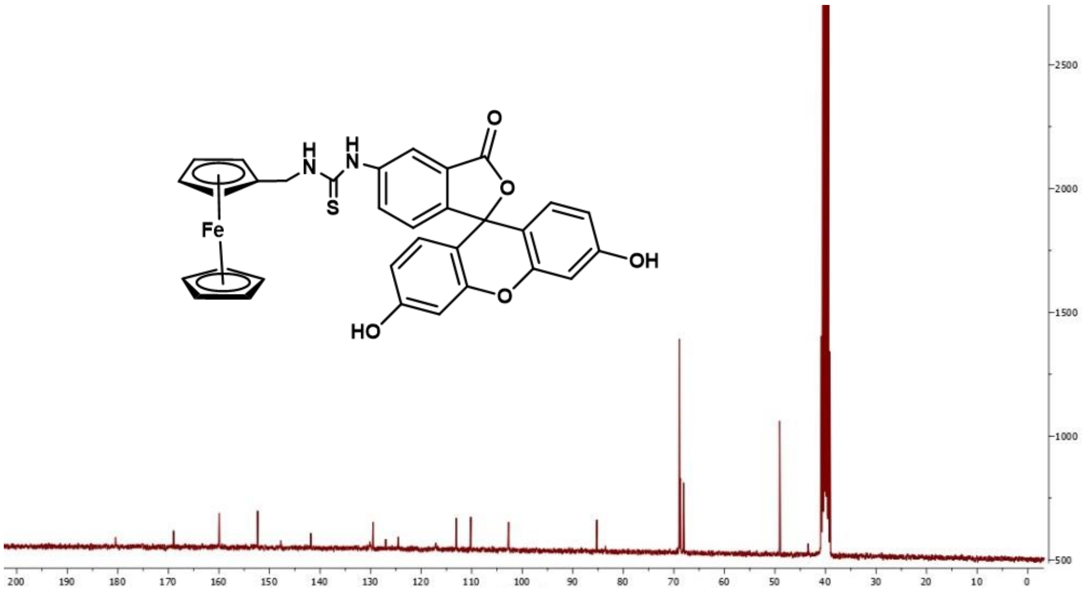

Figure S24: ^{13}C NMR spectrum of ferrocene-fluorescein conjugate (Fc-FITC)

(iii) HRMS of ferrocene-fluorescein conjugate (Fc-FITC)

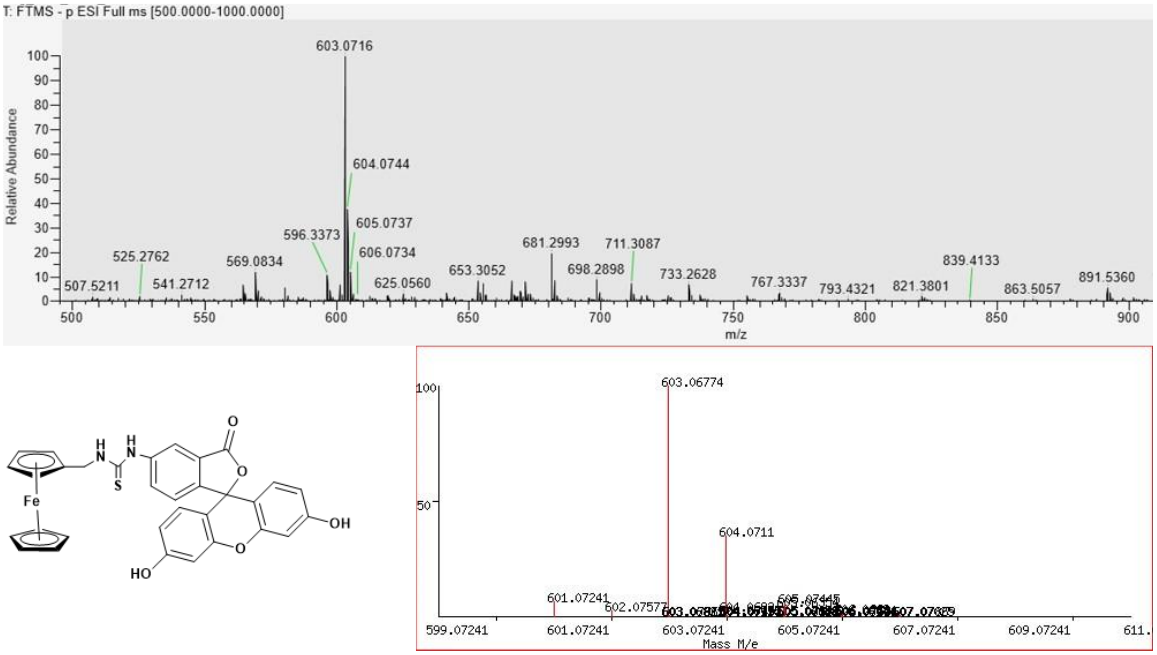

Figure S25: Mass spectrum of Fc-FITC: (a) experimental spectrum in negative ion mode; (b) simulated spectrum of cleaved proton [$FcCH_2$ – H]$^-$ The simulated spectrum reflects the experimentally observed abundant mass and was generated using an online tool: *<u>https://www.sisweb.com/mstools/isotope.html</u>*

S5. Buffer solutions

The aqueous buffers listed below were prepared, and they were used to determine the pKa and to optimize the pH-dependent UV-Vis and fluorescence emission spectroscopy of both FITC and the Fc-FITC conjugate. Identifying these optimal conditions was critical for ensuring the accuracy and stability of subsequent photophysical and photostability analyses.

**Aqueous buffers used**		**Adjusted pH values**
Potassium chloride and hydrochloric acid	KCl/ HCl	2
Acetic acid and sodium acetate	CH_3COOH/ NaOAc	3, 4, 5
Potassium dihydrogen phosphate and sodium hydroxide	KH_2PO4/NaOH	6, 7, 8
Sodium tetraborate decahydrate and hydrochloric acid	$Na_2[B_4O_5(OH)_4]\cdot 8H_2O$/HCl	9, 10, 11
Potassium chloride and sodium hydroxide	KCl/NaOH	12, 13

Table S1: Aqueous 0.1 M buffers and corresponding pH values.

Reference for preparation: <u>http://delloyd.50megs.com/moreinfo/buffers2.html</u>

S6. Photophysical characterization

This section establishes the foundational photophysical properties of the individual components for baseline comparison with the Fc-FITC conjugate.

Fc and FITC individual components

Property	Ferrocene (Fc) [a]	Fluorescein isothiocyanate (FITC)(b)
**Molar Mass**	216.06 g/mol	389.38 g/mol
**Appearance**	Reddish-orange solid	Orange solid
**Molar extinction coefficient (ε)**	λ_{abs} = 322 nm, (ε = 61 $Lmol^{-1}cm^{-1}$) λ_{abs} = 442 nm, (ε = 95 $Lmol^{-1}cm^{-1}$)	λ_{abs} = 495 nm, (ε = 75,000 $Lmol^{-1}cm^{-1}$)
**Emission (λ_{em})**	N/A	λ_{em} = 525 nm,
**Quantum yield (Φ)**	N/A	$\lambda_e\Phi$ ~0.92
**Fluorescence lifetime**		Measured 4.06 ns in EtOH/H_2O, pH 8 buffer Literature values (range from ~3.8–4.1 ns depending on the pH environment
**pH sensitivity**		Strong, the optimal fluorescence at pH ~9
**Photostability**		Moderate and prone to photobleaching
**Prepared $FcCH_2OH$* solution in methanol**	Absorbance 0.128 at 442 nm, **1340** micromolar	Absorbance 0.172 at 495 nm **2.30** micromolar

*Table S2: Properties of Ferrocene (Fc) and Fluorescein Isothiocyanate (FITC). *$FcCH_2OH$ properties were assumed to closely resemble literature values for ferrocene.*

a) Sørensen , T. J., & Nielsen, M. F. (2011). Synthesis, UV/vis spectra and electrochemical characterisation of arylthio and styryl substituted ferrocenes. Central European Journal of Chemistry, 9, 610-618. https://doi.org/10.2478/s11532-011-0040-8
b) https://www.rndsystems.com/products/fitc_5440?utm

Absorption spectra of Fc and FITC

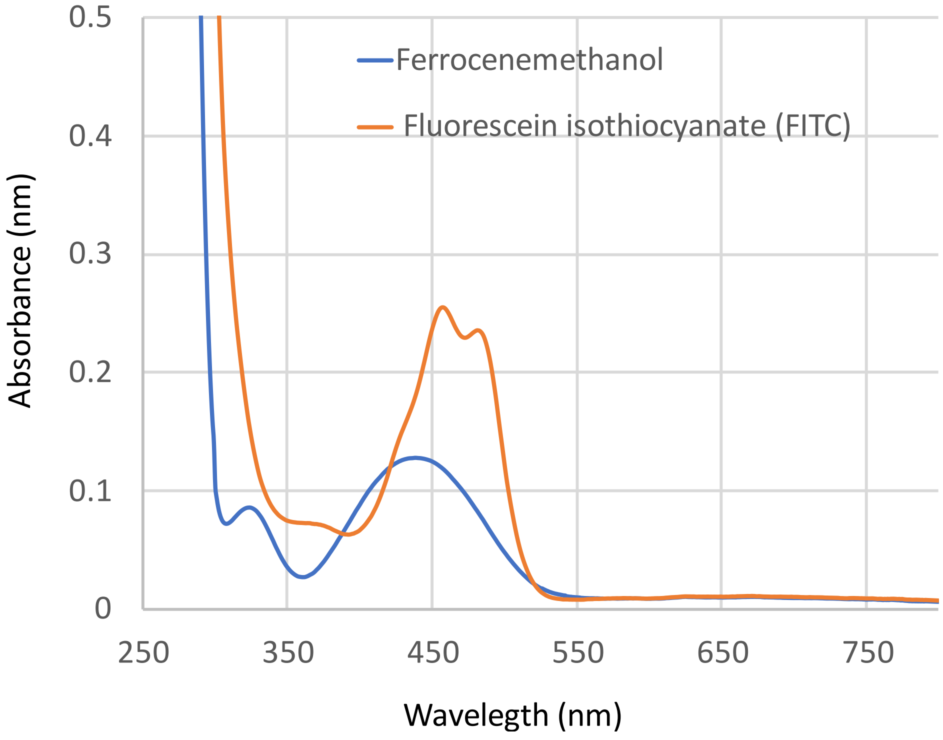

Figure S26: UV–vis absorption of $FcCH_2OH$ and FITC in methanol

Stern–Volmer quenching of FITC by ferrocene derivatives

Fluorescence quenching experiments were performed to quantify the intermolecular interaction between the fluorophore FITC and the ferrocene derivatives (Fc, $FcCH_2OH$, and $FcCH_2NH_2$). Stock solutions of ~2 μM FITC were prepared in a 1:1 $EtOH/H_2O$ buffer (pH 8). Quencher solutions (prepared separately in ethanol) were added incrementally to maintain a constant FITC concentration while varying the quencher concentration ([Q]). Fluorescence emission spectra were recorded at 530 nm (λ_{ex}=500 nm). The Stern–Volmer quenching constants (K_{SV}) were determined by plotting the ratio of unquenched to quenched fluorescence intensity ((F_0/F) against the quencher concentration ([Q]) and fitting the data.

$$F_0/F = 1 + K_{SV}[Q]$$

Where F_0 and F are the fluorescence intensities without and with the quencher [Q], and K_{SV} is the Stern–Volmer quenching constant (M^{-1}). The resulting linear plots (Figure S27) indicate a dynamic quenching mechanism (R^2>0.96). Table S3 below summarizes the calculated K_{SV} values and the corresponding bimolecular quenching rate constants ($k_q = K_{SV}/\tau_0$) assuming FITC lifetime to be (τ_0) of 4.0×10^{-9} s).

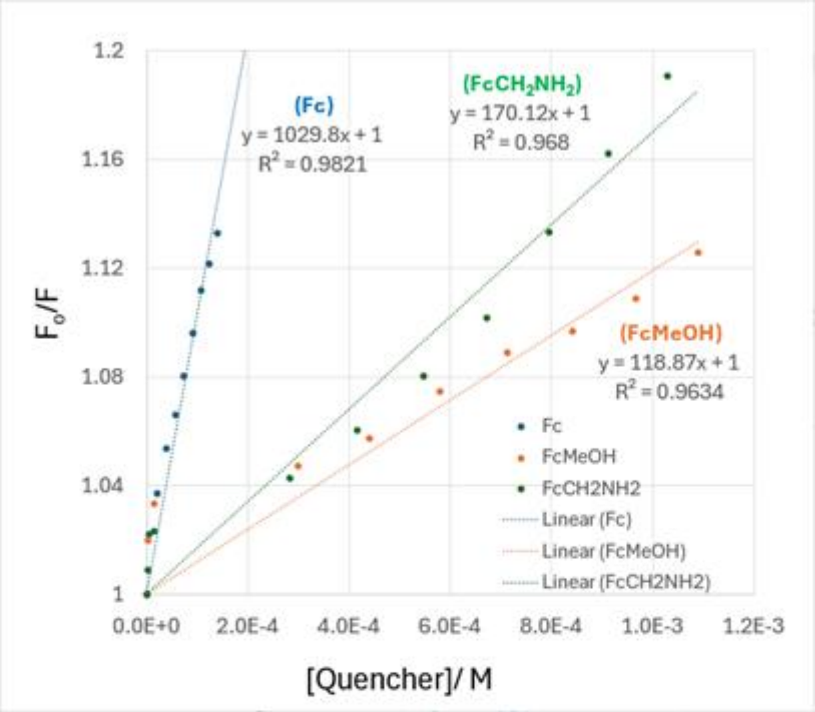

Figure S27: Stern–Volmer plots for the fluorescence quenching of FITC by ferrocene (Fc), $FcCH_2OH$, and $FcCH_2NH_2$ in EtOH/H_2O (1:1 v/v) buffer at pH 8. ***Note****: The Fc plot uses a reduced concentration range, reflecting the Fc moiety's higher inherent quenching efficiency, which yielded a strong linear response (R^2>0.96) at lower concentrations.*

Quencher [Q]	K_{SV} (M^{-1})	τ_0 (s)	kq ($M^{-1}s^{-1}$)	Relative quenching efficiency (%)
Ferrocene (Fc)	1029.8	4.1 x 10 $^{-09}$	2.51 x 10^{11}	100% (reference)
$FcCH_2OH$	118.9	4.1 x 10 $^{-09}$	2.90 x 10^{10}	12% relative to Fc
$FcCH_2NH_2$	170.1	4.1 x 10 $^{-09}$	4.15 x 10^{10}	17% relative to Fc

Table S3: Summary of ferrocene derivatives quenching FITC.

Determination of pKa and optimal pH for emission studies of FITC

FITC and Fc-FITC stock solutions in ethanol were prepared, ensuring absorbance at 485 nm was maintained below 0.2 a.u. for accurate spectral measurements. These stock solutions were combined 1:1 (v/v) with buffer solutions (Table S1) to have an EtOH/H_2O (1:1 v/v) mixture for pKa determination and optimum pH measurement. UV-Vis absorption and emission spectra were collected from pH 2 to 13. The pKa value, which represents the equilibrium between the protonated and deprotonated species, was calculated by plotting absorbance or emission intensity at specific wavelengths against pH. The sigmoidal transition midpoint was pKa. These emission intensity versus pH plots were also used to determine the pH where FITC and the Fc-FITC conjugate had the highest fluorescence signal. Quantitative fluorescence-based studies, including quantum yield and photobleaching assessments, were optimized at this pH for enhanced signal stability.

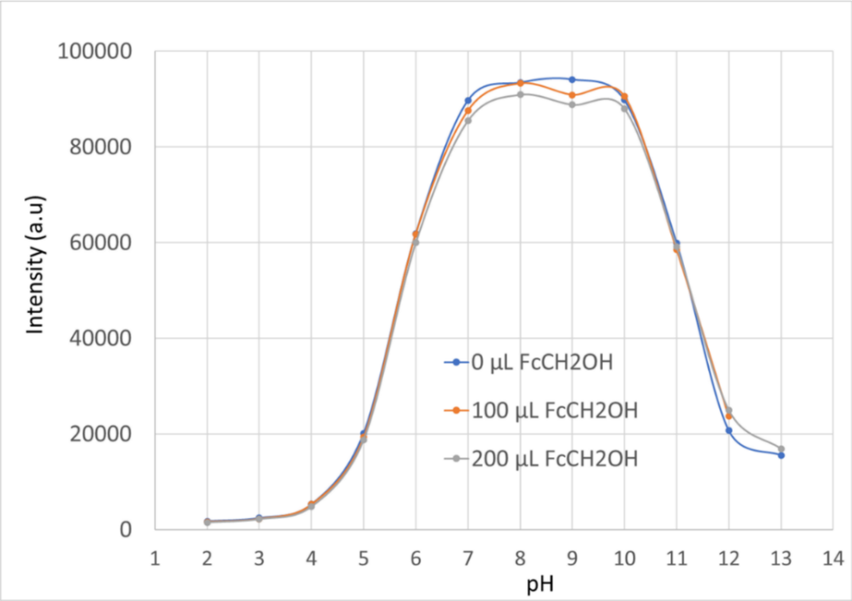

Figure S28: FITC emission intensity (530 nm) vs. pH in EtOH/H_2O (1:1 v/v) buffer following additions of dilute $FcCH_2OH$, illustrating the complex interaction between pH-dependent speciation and intermolecular quenching effects.

Ferrocene-Fluorescein Conjugate (Fc-FITC)

This section shows the photophysical spectra of the synthesized Fc-FITC conjugate in methanol before full optimization in buffered aqueous solution. The figure confirms that the fluorescein chromophore's characteristic optical signature is retained after conjugation. The emission maximum at 525 nm and the excitation maximum near 500 nm are characteristic features of the FITC moiety.

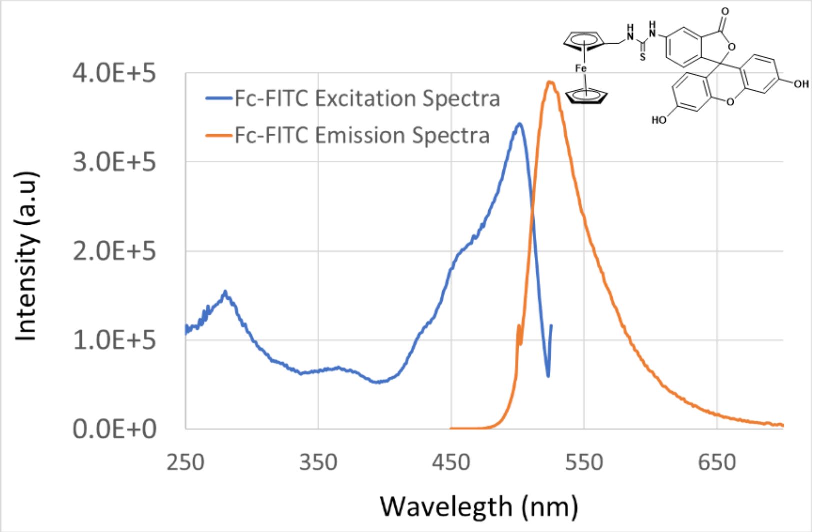

Figure S29: Fc-FITC excitation (blue monitored at 525 nm) and emission (orange, excited at 500 nm) spectra in methanol

Quantum yield (QY) calculations (Fc-FITC)

The fluorescence quantum yield (Φ_S) of Fc-FITC conjugate was determined relative to FITC (Φ_R =0.92) by the comparative method. The QY was determined using the following equation:

$$Q_S = Q_R \text{ x } \frac{I_S}{I_R} \text{ x } \frac{A_R}{A_S} \text{ x } \frac{\eta_S^2}{\eta_R^2}$$

Where subscripts (S) and (R) denote the sample (Fc-FITC) and the reference (FITC), respectively. (I) denotes the integrated fluorescence emission area (450–700 nm), (A) represents the absorbance at the excitation wavelength (500 nm), and (η) signifies the refractive index of the solvent. Since both the sample and reference were measured in the identical solvent mixture (EtOH/H_2O, 1:1 v/v, η = 1.344) at pH 8, the refractive index ratio ($\frac{\eta_S^2}{\eta_R^2}$ = 1) cancels out. Using the integrated emission areas and absorbance values, the QY of Fc-FITC was determined to be Φ=0.17.

Fluorescence lifetime measurement of Fc-FITC.

Fluorescence lifetime measurements were conducted using the FLS 1000 Photoluminescence Spectrometer equipped with a pulsed LED laser (λ_{ex}=247 nm). The resulting decay profiles were recorded, and the instrumental response function was deconvoluted from the data. The intensities were presented on a linear scale, and the decay curves (Figure S30 is from the instrument) were analyzed by fitting to a single exponential decay function. To ensure a valid comparison, the lifetime of unconjugated FITC was also measured under identical conditions (EtOH/H_2O 1:1 v/v, pH 8). The precise experimental value obtained for FITC was τ_0 = 4.06 ns. For consistency and clear presentation, this value was approximated as 4.1 ns for calculations within the main text.

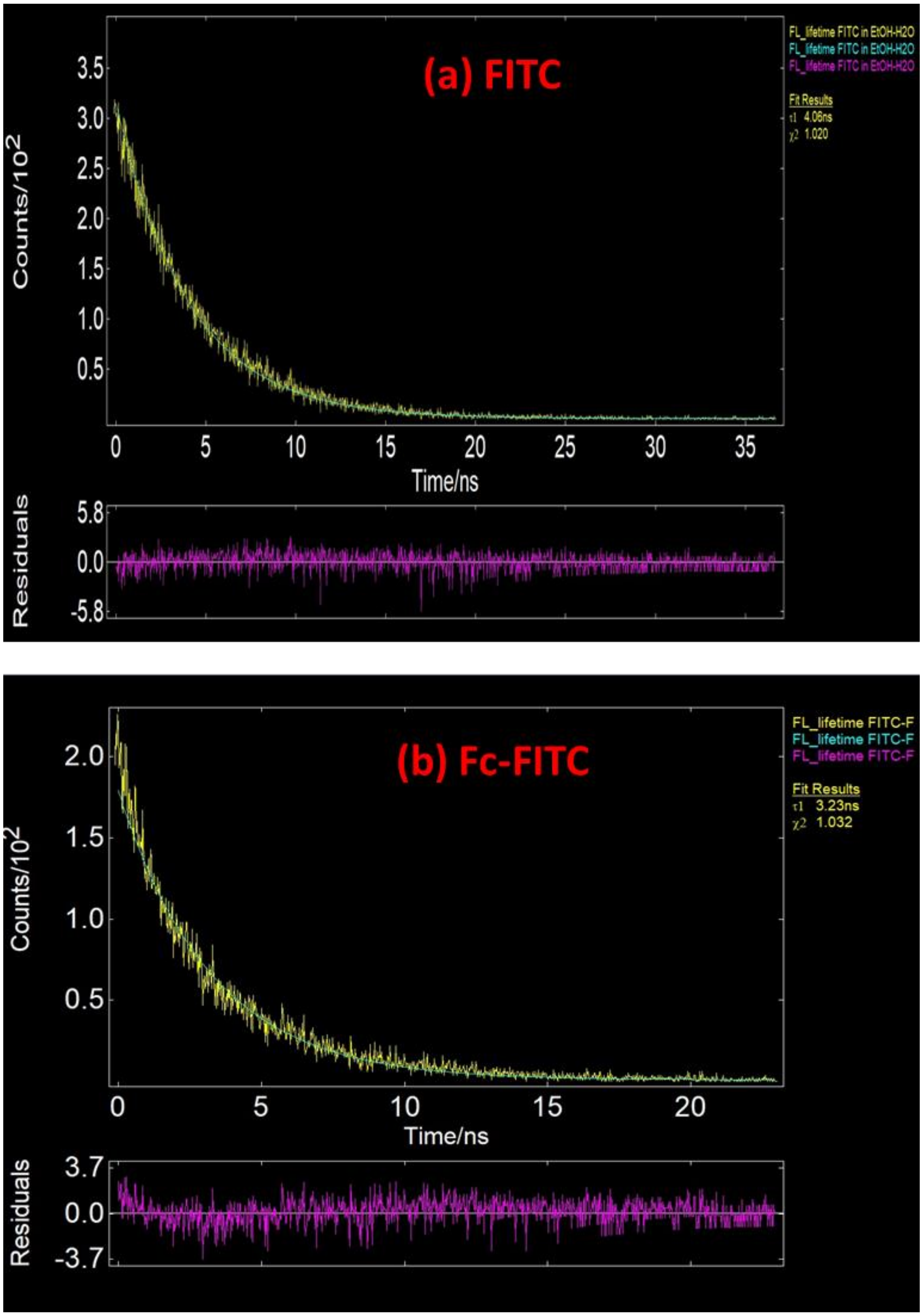

Figure S30: Representative fluorescence decay plots (from instrument) of (a) FITC and (b) Fc-FITC in EtOH/H_2O (1:1 v/v) at pH 8 (λ_{ex}=247 nm).

S7. Computational studies

Computational methods were employed to offer theoretical information about the electronic structure, spectral properties, and PET mechanism of the Fc-FITC conjugate.

Given that FITC exists as a multivalent anion at the experimental pH of 8–9 (with both the carboxyl and phenolic groups deprotonated), deprotonated forms of FITC and Fc-FITC were used in all computational calculations. Geometries for both compounds were first fully optimized to determine the lowest energy ground-state conformations (Figure S31). Electronic structure and UV-Vis absorption spectra were simulated using Time-Dependent Density Functional Theory (TDDFT). Calculations explored two functionals, B3LYP[1–4] and CAM-B3LYP[5] and two environmental conditions, the gas phase and the SMD implicit solvation model[6] (representing the EtOH/H_2O buffer). A custom basis set (cc-pVDZ, with LANL2DZ for metal atoms) was used. All computations were performed using the Gaussian16 software[7].

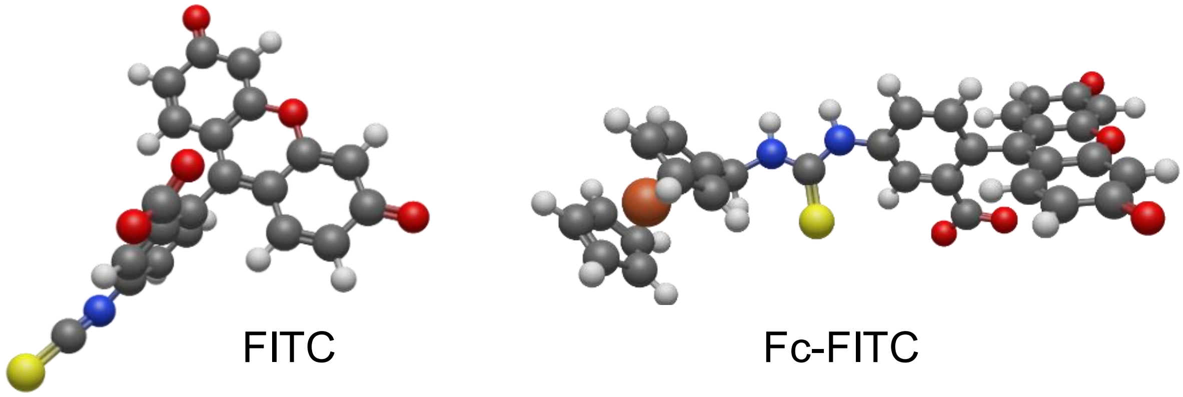

Figure S31. Optimized ground-state conformations of the deprotonated FITC (left) and Fc-FITC (right) molecules, as modeled/ optimized for computational calculations.

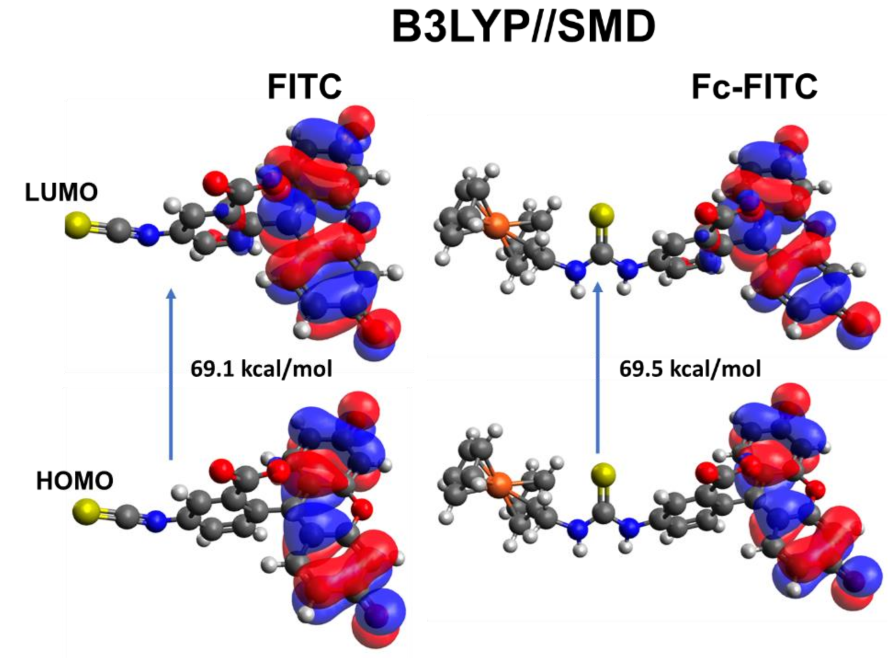

Figure S32. DFT predictions of the frontier molecular orbitals (HOMO and LUMO) for FITC (left) and Fc-FITC (right) using the B3LYP//SMD method.

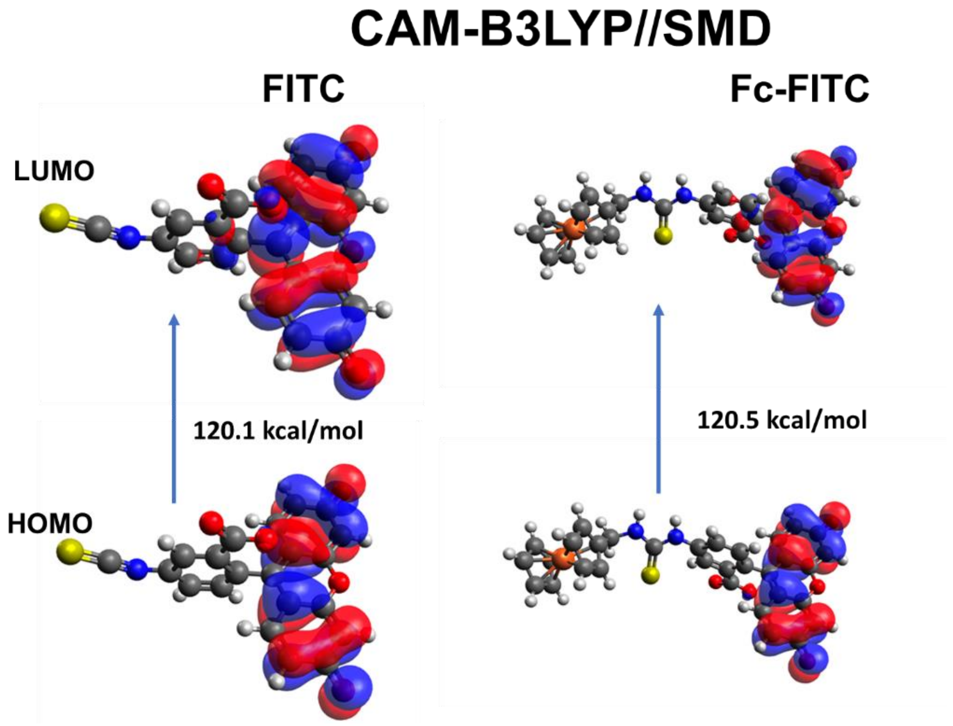

Figure S33. DFT predictions of the frontier molecular orbitals (HOMO and LUMO) for FITC (left) and Fc-FITC (right) using the CAM-B3LYP//SMD method.

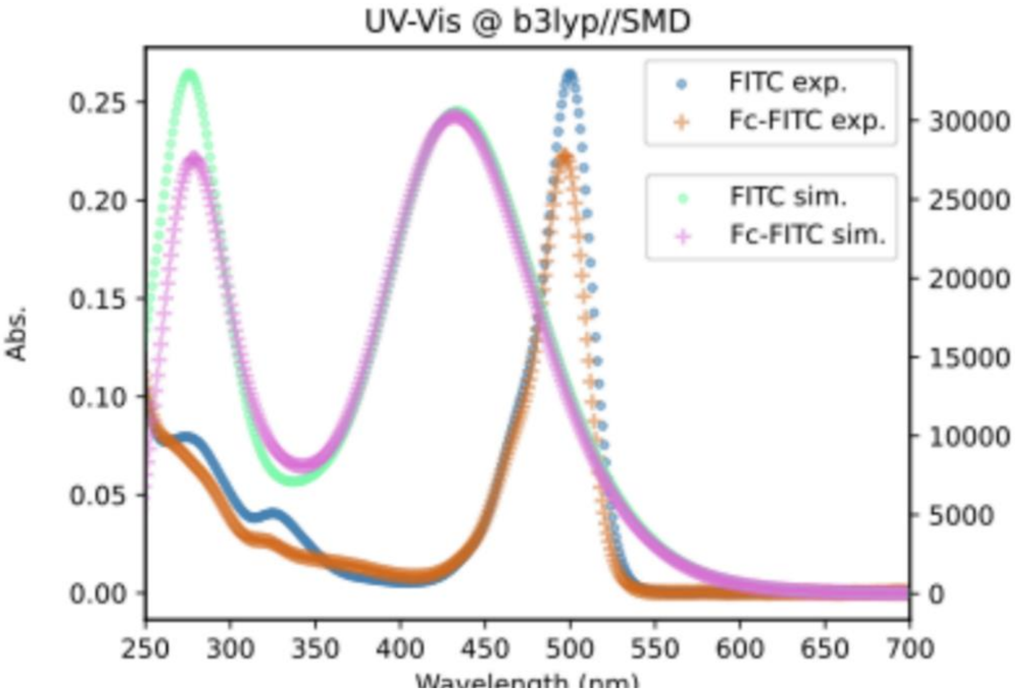

Figure S34. Comparative experimental and computational UV-Vis absorption using the B3LYP//SMD method.

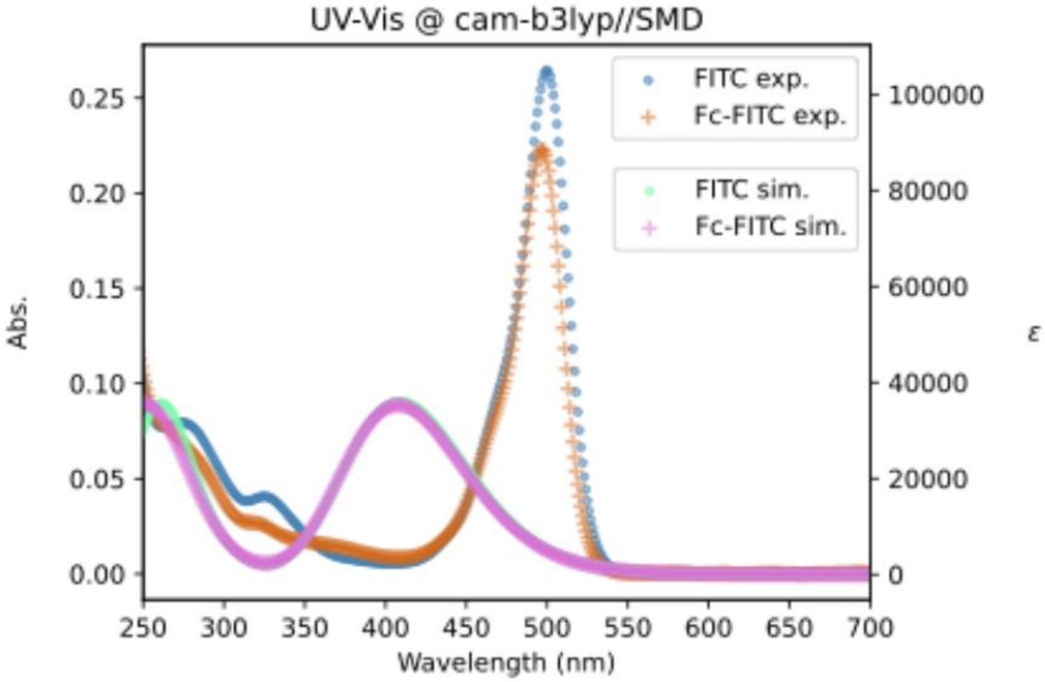

Figure S35. Comparative experimental and computational UV-Vis absorption using the CAM-B3LYP//SMD method.

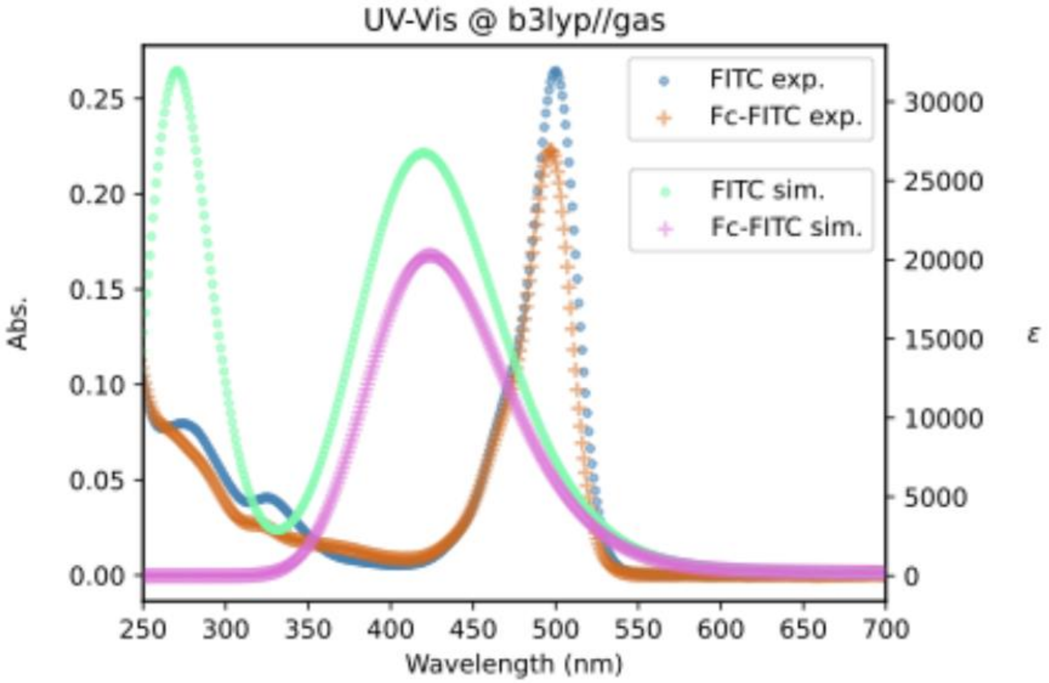

Figure S36. Comparative experimental and computational UV-Vis absorption using the B3LYP//gas method

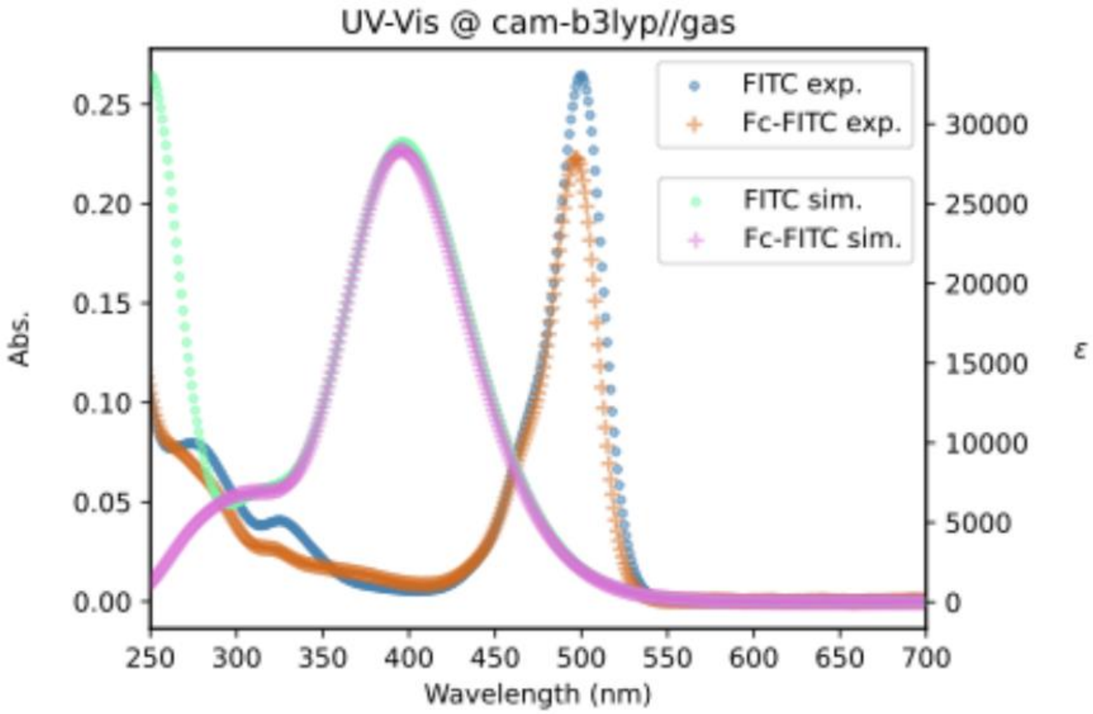

Figure S37. Comparative experimental and computational UV-Vis absorption using the CAM-B3LYP//gas method.

S8. Photostability studies

This section quantifies the enhanced photostability of the Fc-FITC conjugate under continuous high-intensity illumination. FITC and the Fc-FITC conjugate photobleaching kinetics were analyzed by monitoring fluorescence intensity at 530 nm over 60 minutes of continuous UV-Vis irradiation (320–500 nm) at 23 mW/cm2 (Figure S38). A first-order exponential model was used to fit decay data $I(t) = I_0 \cdot e^{-Kt}$, where I_0 represents the initial fluorescence intensity and K is the photobleaching rate constant and $t_{1/2} = \ln(2)/k$ represents the photobleaching half-life. Fc-FITC photostability improved significantly, as seen in Table S4.

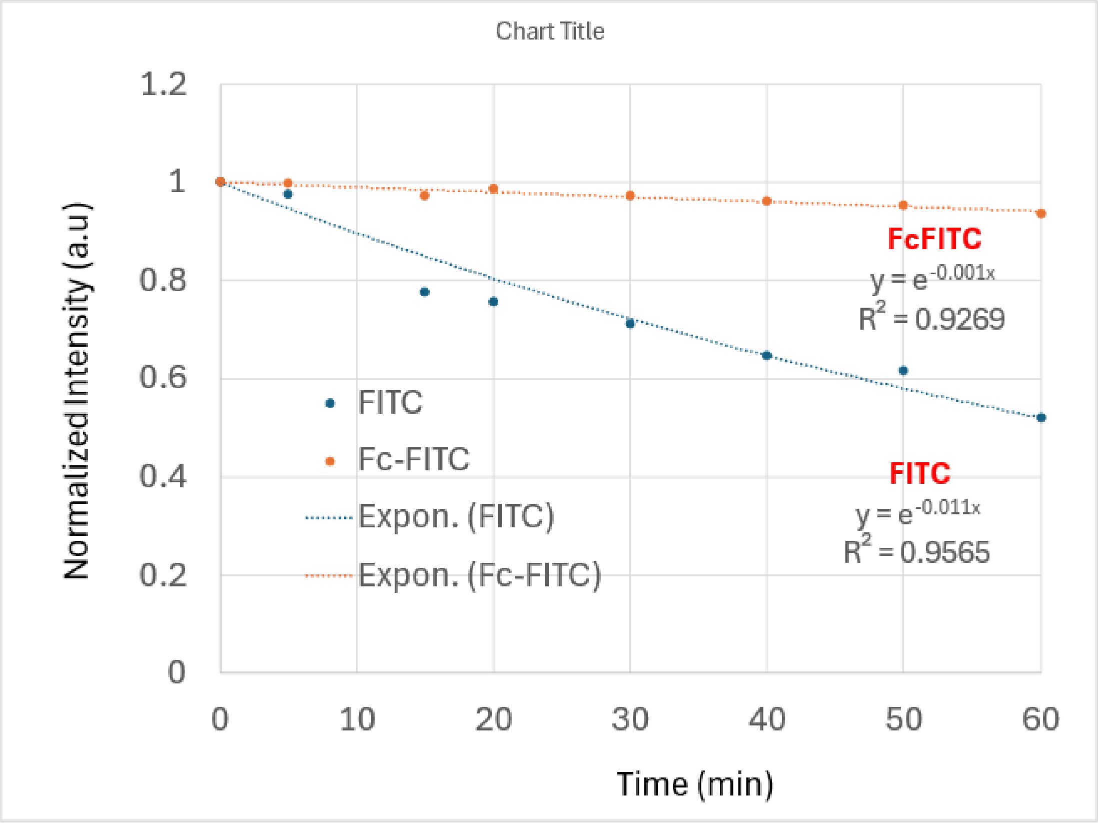

Figure S38: Photobleaching kinetics of FITC and Fc-FITC fitted to a first-order exponential decay model, illustrating the dramatic difference in their functional lifetimes.

**Fluorophore**	**Decay constant k (min^{-1})**	**Half-life $t_{1/2} = \ln(2)/k$ (min)**
FITC	≈ 0.011	≈ 63.01 minutes
Fc-FITC	≈ 0.001	≈ 693.15 minutes

Table S4. Photobleaching rate constants and half-lives of FITC and Fc-FITC under continuous UV-Vis irradiation (320–500 nm, 23 mW/cm²) in EtOH/H_2O (1:1, pH 8).

Qualitative visualization of photoprotection

Direct visual comparison of solutions supports the quantitative data. Figure S39 visually illustrates the difference in degradation between the two samples after 60 minutes of high-intensity irradiation.

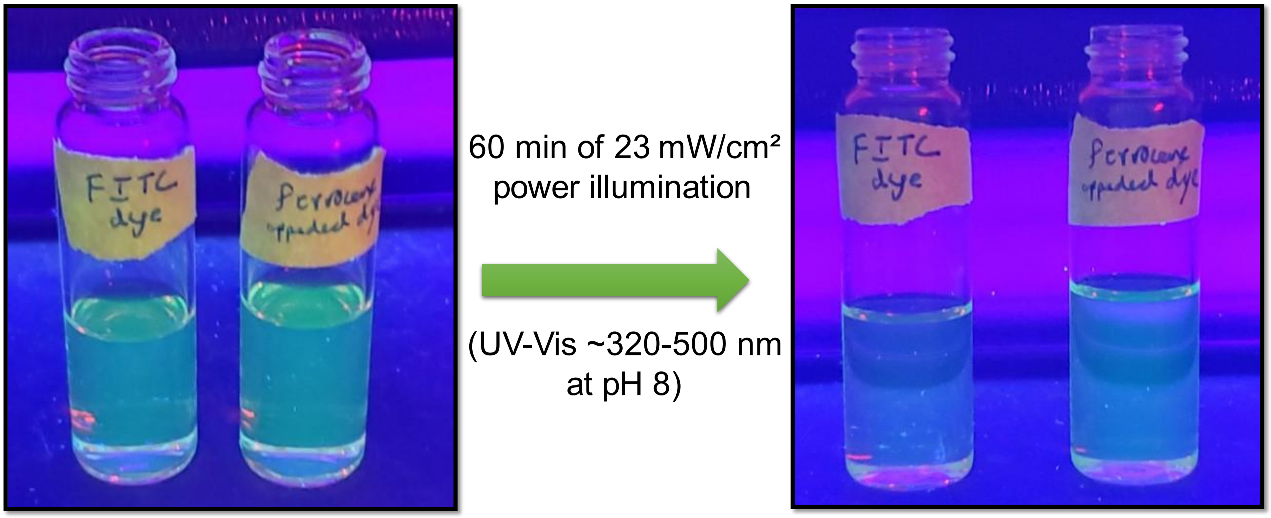

Figure S39. Visual comparison of FITC and Fc-FITC solutions before (left) and after (right) 60 minutes of UV-Vis irradiation (320-500 nm, 23 mW/cm²) at pH 8.

S9. Singlet oxygen ($^1O_2$) generation assay

Singlet Oxygen Sensor Green (SOSG, Invitrogen) was prepared as a 5 mM stock solution in methanol and stored at −20°C. Working solutions were diluted to 2 µM in a pH 8.0 buffer (EtOH/H_2O, 1:1 v/v). To ensure equal photon absorption across all comparative sets, FITC and Fc–FITC concentrations were adjusted to yield an identical absorbance at the irradiation peak wavelength (A_{505} = 0.115).

Samples (6 mL) were placed in 20 mL borosilicate glass scintillation vials under constant stirring and irradiated using an OmniCure S1500 light source equipped with a 320–500 nm band-pass 23 mW/cm² irradiation. Fluorescence emission spectra (λ_{ex} = 505 nm, λ_{em} = 500 - 700 nm) were collected at t = 0, 2.5, 5, 10, 15, 20, 25, and 30 minutes.

To isolate the signal generated by the SOSG end-product from the intrinsic emission and photobleaching of the fluorescein core, dye-only controls (without SOSG) were recorded under identical conditions. A background-subtraction protocol was implemented:

$$F_{\text{corrected}}(t) = F_{(\text{Dye + SOSG})}(t) - F_{(\text{Dye only})}(t)$$

The net increase in SOSG fluorescence was computed as

$$\Delta F(t) = F_{\text{corrected}}(t) - F_{\text{corrected}}(0)$$

The apparent singlet oxygen generation rate was determined via linear regression of the 0–30 min region.

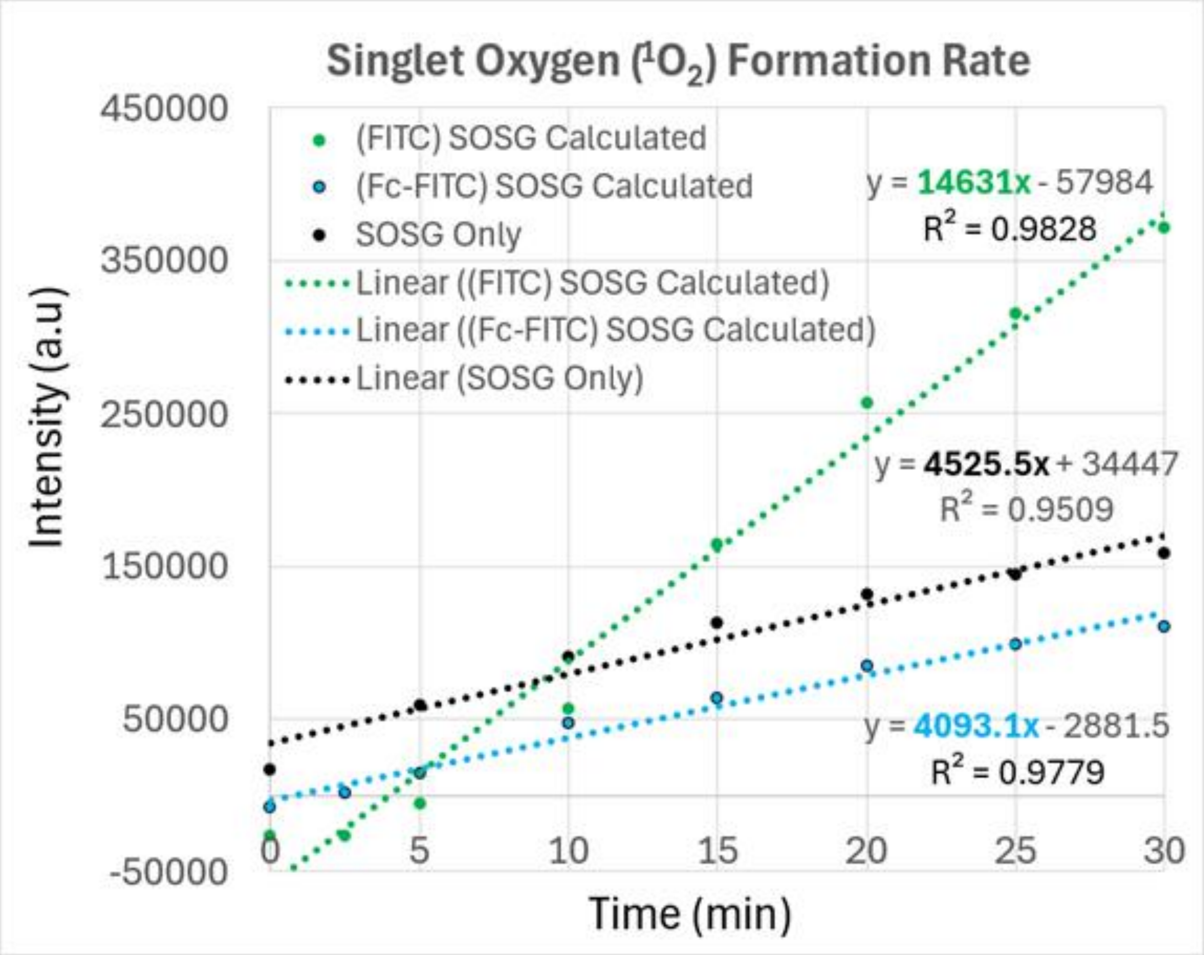

Figure S40. Corrected $^1O_2$ generation kinetics. Background-subtracted SOSG fluorescence (ΔF at 535 nm) for FITC (green), Fc–FITC (blue), and control (black) under continuous UV-Vis irradiation (320-500 nm, 23 mW/cm²). Conditions: pH 8.0 EtOH/H_2O (1:1), matched A_{505} = 0.115. Dashed lines indicate linear fits (0–30 min).

Sample	***Rate (k)*** **(a.u./min^1)**	**Relative $^1O_2$ Generation (%)**	**NaN_3 Quenching (%)**
FITC + SOSG	1.46×10^4	100	32.5
Fc–FITC + SOSG	4.09×10^3	28	4.6
SOSG only	4.53×10^3	32	5.1

Table S5. $^1O_2$ generation and NaN_3 quenching parameters for FITC and Fc–FITC (A_{505} = 0.115).

Mechanistic validation (Quencher controls)

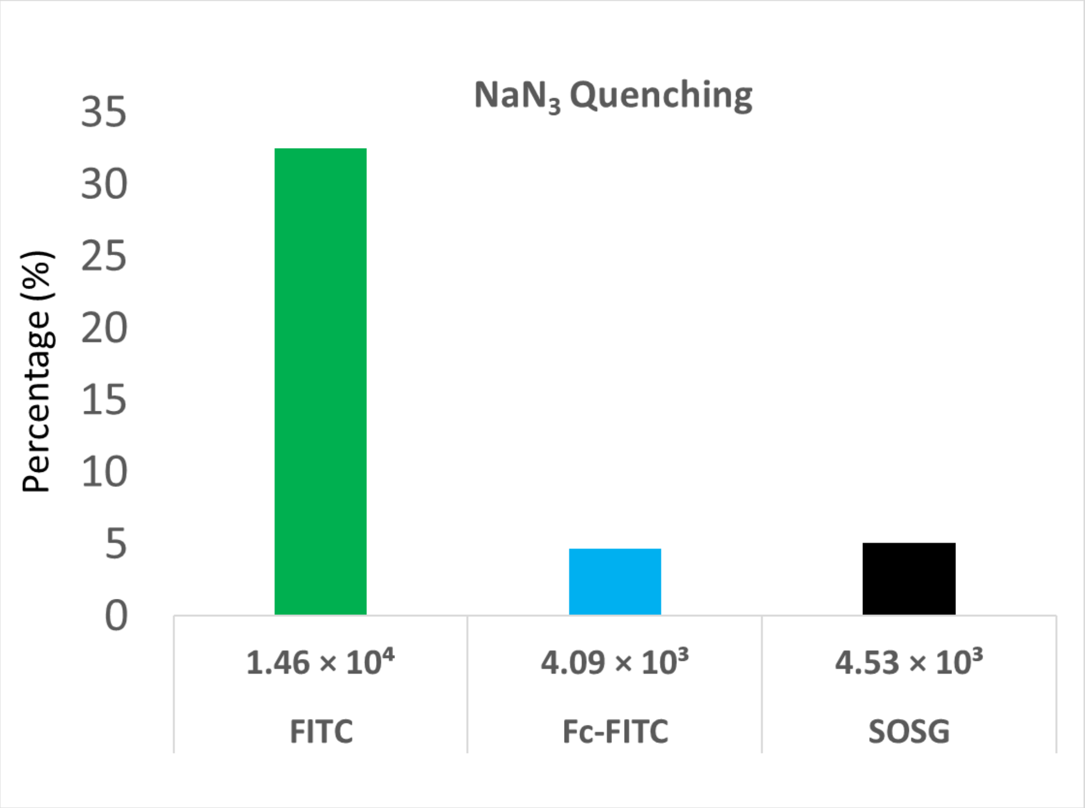

Figure S41. Sensitization rates and NaN_3 quenching analysis (bottom). Apparent $^1O_2$ generation rates (vertical), emission quenching (%) following 10 mM NaN_3 addition. Substantial FITC quenching (32.5%) versus minimal Fc–FITC quenching (4.6%) validates PET-mediated suppression of triplet-state accessibility.

The specificity of the assay for $^1O_2$ was verified by the addition of sodium azide (NaN_3, 10 mM) after 30 minutes of irradiation. The resulting percentage of emission quenching was calculated relative to the t = 30 min intensity. Dark controls (wrapped in foil) and SOSG controls (no dye) were monitored to account for spontaneous oxidation and probe self-sensitization, respectively.

References

1. Becke, A. D. Density-functional thermochemistry. III. The role of exact exchange. *J. Chem. Phys.* **98**, 5648–5652 (1993).
2. Lee, C., Yang, W. & Parr, R. G. Development of the Colle-Salvetti correlation-energy formula into a functional of the electron density. *Phys. Rev. B* **37**, 785–789 (1988).
3. Vosko, S. H., Wilk, L. & Nusair, M. Accurate spin-dependent electron liquid correlation energies for local spin density calculations: a critical analysis. *Can. J. Phys.* **58**, 1200–1211 (1980).
4. Stephens, P. J., Devlin, F. J., Chabalowski, C. F. & Frisch, M. J. Ab Initio Calculation of Vibrational Absorption and Circular Dichroism Spectra Using Density Functional Force Fields. *J. Phys. Chem.* **98**, 11623–11627 (1994).
5. Yanai, T., Tew, D. P. & Handy, N. C. A new hybrid exchange–correlation functional using the Coulomb-attenuating method (CAM-B3LYP). *Chem. Phys. Lett.* **393**, 51–57 (2004).
6. Marenich, A. V., Cramer, C. J. & Truhlar, D. G. Universal Solvation Model Based on Solute Electron Density and on a Continuum Model of the Solvent Defined by the Bulk Dielectric Constant and Atomic Surface Tensions. *J. Phys. Chem. B* **113**, 6378–6396 (2009).
7. M. J. Frisch, G. W. Trucks, H. B. Schlegel, G. E. Scuseria, M. A. Robb, J. R. Cheeseman, G. Scalmani, V. Barone, G. A. Petersson, H. Nakatsuji, X. Li, M. Caricato, A. V. Marenich, J. Bloino, B. G. Janesko, R. Gomperts, B. Mennucci, H. P. Hratchian, J. V. Ortiz, A. F. Izmaylov, J. L. Sonnenberg, D. Williams-Young, F. Ding, F. Lipparini, F. Egidi, J. Goings, B. Peng, A. Petrone, T. Henderson, D. Ranasinghe, V. G. Zakrzewski, J. Gao, N. Rega, G. Zheng, W. Liang, M. Hada, M. Ehara, K. Toyota, R. Fukuda, J. Hasegawa, M. Ishida, T. Nakajima, Y. Honda, O. Kitao, H. Nakai, T. Vreven, K. Throssell, J. A. Montgomery, Jr., J. E. Peralta, F. Ogliaro, M. J. Bearpark, J. J. Heyd, E. N. Brothers, K. N. Kudin, V. N. Staroverov, T. A. Keith, R. Kobayashi, J. Normand, K. Raghavachari, A. P. Rendell, J. C. Burant, S. S. Iyengar, J. Tomasi, M. Cossi, J. M. Millam, M. Klene, C. Adamo, R. Cammi, J. W. Ochterski, R. L. Martin, K. Morokuma, O. Farkas, J. B. Foresman, and D. J. Fox. Gaussian16 Revision C.02. Gaussian Inc. (2016).
